# Extremely low genetic diversity in a circumpolar dragonfly species, *Somatochlora sahlbergi* (Insecta: Odonata: Anisoptera)

**DOI:** 10.1038/s41598-018-32365-7

**Published:** 2018-10-11

**Authors:** Manpreet K. Kohli, Göran Sahlén, William R. Kuhn, Jessica L. Ware

**Affiliations:** 1 Department of Biological Sciences, Rutgers University-Newark, Newark, USA; 2 Ecology and Environmental Science, RLAS, Halmstad University, Halmstad, Sweden; 3 Department of Electrical Engineering and Computer Science, University of Tennessee, Knoxville, USA

## Abstract

We present the first empirical treatment of the northernmost breeding dragonfly, *Somatochlora sahlbergi*. We sequenced populations from United States, Canada, Finland, Sweden and Norway for cytochrome oxidase I (COI) and D2 region of 28s. We found that, despite geographic barriers across its vast arctic range, *S. sahlbergi* is a single species. Not only does it appear to interbreed across its entire range, there also seems to be almost no variation among European and North American populations in their COI gene fragment (the barcode gene), which is usually extremely variable. We further found that characters thought to be diagnostic for the larvae of *S. sahlbergi* were absent in our European samples. We review and re-describe the habitat of this species based on new findings from recent field observations. Finally, we report for the first time the likely presence of this species in Japan. We hope our findings will encourage further study of this species and other under-studied insect taxa that inhabit the remote Arctic.

## Introduction

**Figure 1:**
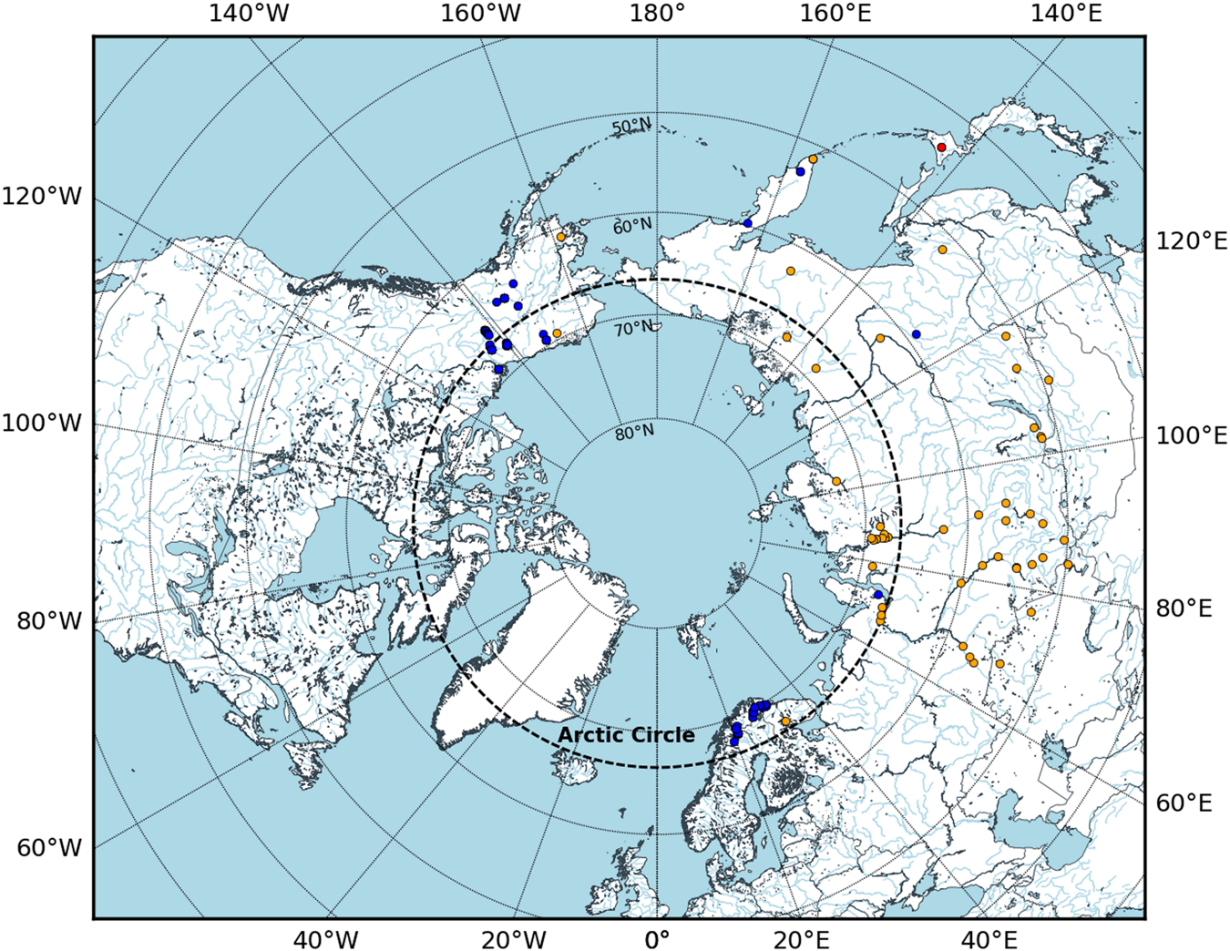
Holarctic distribution of *S. sahlbergi* — locations with exact coordinates are shown in blue, while locations estimated from literature are shown in orange. The approximate location of a novel potential population of *S. sahlbergi* in Japan (informed by NCBI data), is shown in red. See Supp. Table S1 for record details.

*Somatochlora sahlbergi* Trybom is an enigmatic dragonfly that is distributed at high latitudes across the Holarctic (see Fig. 1). First described by F. Trybom in 1889, due to its extreme habitat this species remains understudied. Adults of this species are mid-sized, dark metallic green dragonflies, much like other members of the genus *Somatochlora*. They have hyaline wings and a dense coat of setae on their body^1,2^. The shape of the cerci in the male is distinctive from other members of this genus^2^. The larva is dark reddish brown or orange-brown with a relatively pale and yellowish venter^3^. Like the adult, the larva also has a dense coat of setae. Larvae are suggested to have long lateral spines on abdominal segments 8 and 9^4^; however, this character has not been well assessed across the geographic range of the taxon.

*Somatochlora sahlbergi* has the northernmost breeding range of any of the world’s approximately 6000 odonate species^5–7^. The southern limit of most of its populations in Europe and North America is at higher latitudes than the northern limits of most Holarctic dragonfly species. The species has been reported from about 71 locations across Russia, Norway, Finland, Sweden, Canada and the USA (Fig. 1, Supp. Table S1, also see^8^). The southernmost record in North America is at 63°N in western Alaska; the range continues east into the Yukon and Northwest Territories of Canada but as yet no population has been recorded east of the Mackenzie River^3^. Donnelly^9^ reports a single exception from 68.75°N 134.25°W (OdonataCentral.org record no. 247913), which has not been confirmed as a *S. sahlbergi* locale by the present authors (this location is probably a regional centroid rather than an exact locality). In Europe, the southernmost limit is 68°N, across Finland, Norway and Sweden. In Asia, this species occurs farther south than in North America and Europe, extending to 51.65°N along the Mongolian border and in the Kamchatka Peninsula of Russia. Although *S. sahlbergi* has relatively restricted distribution in North America and Europe, in Russia according to Belyshev^10^, it ranges throughout the Siberia. He recorded populations along the Yana, Indigirka, and Yenisey Rivers and farther south along the Amur and Shilka Rivers, and at Lake Baikal close to the Mongolian border. Additionally, there are populations along the rivers Ob and Lena that transect Russia from north to south.

*S. sahlbergi* is usually associated with peatlands in permafrost (specifically in Canada, Finland, Sweden and Norway), which have low mean annual temperatures and precipitation^3,7,8,11^. Adults fly around these peatlands where their larvae live in cold, deep^3^ and flowing water^10^ (*pers. obs*. MKK and WRK). In most of its North American and European range, *S. sahlbergi* occurs at the latitudinal treeline^3^, north of which trees are unable to grow due to low moisture and extreme temperature. Schröter^8^ suggested that *S. sahlbergi* is closely associated with palsa mires in the region of Fennoscandia (i.e., the Scandinavian Peninsula, Finland, Karelia, and the Kola Peninsula). In Canada and Alaska, larvae are found in deep, cold ponds surrounded by sedges and aquatic mosses and underlain with permafrost soil^7^. These locations are also characterized by annual mean temperatures of less than 0 °C and mean annual rainfall <450 mm^7^.

Although it was described 120 years ago, *S. sahlbergi* is still relatively rare in collections^3,12–16^ and information about its evolutionary history and ecology is poor^8^. It is a fascinating species with a broad and perplexing distribution in some of the planet’s most extreme habitats. In this study, we explore the population structure of this species across its range. We believe that because of its geography *S. sahlbergi* might be isolated into Old World and New World clades without gene flow instead of a single panmictic population. We test the hypothesis that there is distinct genetic structure among populations in North America and Eurasia. In addition, we discuss the utility of lateral spines for taxonomic identification of northern congeneric *Somatochlora* and speculate how the natural history of *S. sahlbergi* may have been influenced by Quaternary glaciation, as is the case with many other northern species.

## Methods

### Taxon Sampling

Specimens were collected in Scandinavia and Finland in September 2014 (Sweden, Norway, Finland, by JLW and GS) and July 2015 (Finland, by Magnus Billqvist). Samples from the Yukon Territory in Canada were collected in August 2015 (by MKK and WRK). In Scandinavia and Finland, we visited known *S. sahlbergi* sites, and also explored potential habitat (see Fig. 1). During the Yukon Territory sampling, we visited all the previously known localities^7^ (see Fig. 1). At each location, we sampled both larvae and adults and collected 28 samples in total that we identified as *S. sahlbergi* from Europe and Canada. Four samples were adults from the Yukon and the rest were larvae from Europe. To supplement our North American sampling, we borrowed 6 North American specimens from the Beaty Biodiversity Museum, University of British Columbia, Vancouver, bringing our specimen count to 34. Besides *S. sahlbergi*, we also collected *Somatochlora albicincta* (Burmeister), which were included in our analysis. See Supp. Table S2 for further information on the samples.

### DNA isolation, gene selection and PCR amplifications

DNA was extracted from legs of the 34 *S. sahlbergi* samples using a Qiagen Blood and Tissue Extraction Kit (Qiagen Sciences, Germantown, MD). We followed the protocol specified by the manufacturer but extended the incubation time to 24 h. Each sample was amplified for the mitochondrial gene cytochrome oxidase I (COI) and a fragment of the nuclear ribosomal 28 S subunit, D2. We used a COI primer pair (Supp. Table S3) which amplifies approximately 516 bp fragment found 214 bp downstream of 5′ region of COI gene. COI, universally regarded as the barcode gene, is a fast-evolving gene that has been repeatedly demonstrated to vary among individuals in a population (see^17–20^). Therefore, this gene fragment should provide enough resolution to discern the genetic structure among the populations. The nuclear gene D2 was chosen to provide an independent dataset to compare to the mitochondrial COI. It contains five hypervariable stem and loop regions, which have been shown to be useful for distinguishing inter- and intrageneric species relationships^21^. The amplified PCR product was purified and sequenced by Macrogen (Macrogen USA, Rockville, MD). We successfully amplified 28 sequences for COI and 25 for D2. All sequences were deposited in the NCBI GenBank database (see Supp. Table S2 for accession numbers).

### Sequence alignment

Initial alignments were performed in ClustalX^22^ and then manually checked for incongruencies in Mesquite^23^. The D2 region was largely invariant among the species in our dataset, hence secondary structural alignment was not necessary. We created two data matrices: one for each of the genes. The final COI matrix consisted of 28 samples sequenced *de novo* and an additional 20 samples amplified at the Environmental Protection Agency (EPA) in Cincinnati, OH (E. Pilgrim, unpublished) bringing our *S. sahlbergi* total to 48 (Supp. Table S2). To determine whether *S. sahlbergi* is a monophyletic species, we included sequences from other *Somatochlora* species, *S. alpestris* (Selys), *S. arctica* (Zetterstedt), *S. clavata* Oguma, *S. dido* Needham, *S. elongata* (Scudder), *S. exuberata* Bartenev, *S. franklini* (Selys), *S. graeseri* Selys, *S. hudsonica* (Hagen), *S. metallica* (Vander Linden), *S. minor* Calvert, *S. semicircularis* (Selys), *S. septentrionalis* (Hagen), *S. uchidai* Förster, *S. viridiaenea* (Uhler), *S. whitehousei* Walker, *S. williamsoni* Walker, and several unknown *Somatochlora* specimens (Supp. Table S2). These sequences were downloaded from GenBank. *Helocordulia uhleri* (Selys) and *Cordulia amurensis* Selys were used as more distant outgroups.

Our final D2 matrix included a total of 42 individuals, of which 25 were *S. sahlbergi* and the rest were *S. albicincta* (Supp. Table S2). This table includes only those specimens that were successfully sequenced (at the Ware Lab) for both genes.

To compare *S. sahlbergi* to other northern odonate species, we downloaded nucleotide sequences from the Barcode of Life Data System^24^ for dragonflies with similar ranges to *S. sahlbergi* in North America and Europe. We included 10 species in addition to *S. albicincta* and *S. sahlbergi* (Supp. Table S4): *Aeshna canadensis* Walker, *A. eremita* Scudder, *A. interrupta* Walker, *A. juncea* (Linnaeus), *A. septentrionalis* Burmeister, *A. subarctica* Walker, *A. umbrosa* Walker, *Leucorrhinia glacialis* Hagen, *Libellula quadrimaculata* Linnaeus, and *Sympetrum danae* (Sulzer). For each of these species, alignments were created in ClustalX and then manually investigated for ambiguities in Mesquite. All alignment files made in this study are available from the corresponding author upon request.

### Phylogenetic and population analysis

We conducted a phylogenetic analysis to check the validity of *S. sahlbergi* as a monophyletic species within the genus *Somatochlora*. We based our phylogenetic reconstruction of the genus *Somatochlora* on COI for which we had a more exhaustive sequence sampling compared to D2. We also reconstructed the relationships among populations of *S. sahlbergi* using a concatenated matrix of COI and D2 data. Phylogenetic analyses for the datasets (COI and COI + D2) were done using maximum likelihood inference via iq-tree^25^. For the COI + D2 dataset, we partitioned the dataset by gene fragment. Substitution models for both the data sets were determined in iq-tree prior to tree reconstruction^26^. Bootstrap values for reconstructed trees from the two datasets were obtained using the ultrafast bootstrap approach in iq-tree, with 1000 replications^27^. The iq-tree analysis was run on the iq-tree web server^28^.

Haplotype analysis of the COI data for all species were performed in popart^29^ using the minimum-spanning network algorithm^30^. Because our preliminary tree reconstruction suggested that 3 *Somatochlora alpestris* sequences taken from GenBank were *S. sahlbergi*, we included these specimens also in our haplotype reconstruction of the latter species, giving a total of 51 samples.

We used mega (v7.0.14)^31^ for a comparative analysis of intraspecific genetic diversity among the 12 northern odonate species, including *S. sahlbergi*. We estimated the percentage of intraspecific diversity (referred to as PID here after) for each of the species using both Jukes-Cantor (JC) and Kimura-2-parameter (K2P) nucleotide evolution models.

### Larval morphology

Larvae were inspected and identified under a Nikon SMZ-745T zoom stereo dissecting microscope. We counted the number of *S. sahlbergi* specimens with and without lateral spines on abdominal segments 8 and 9.

## Results

### Phylogenetic reconstruction

**Figure 2:**
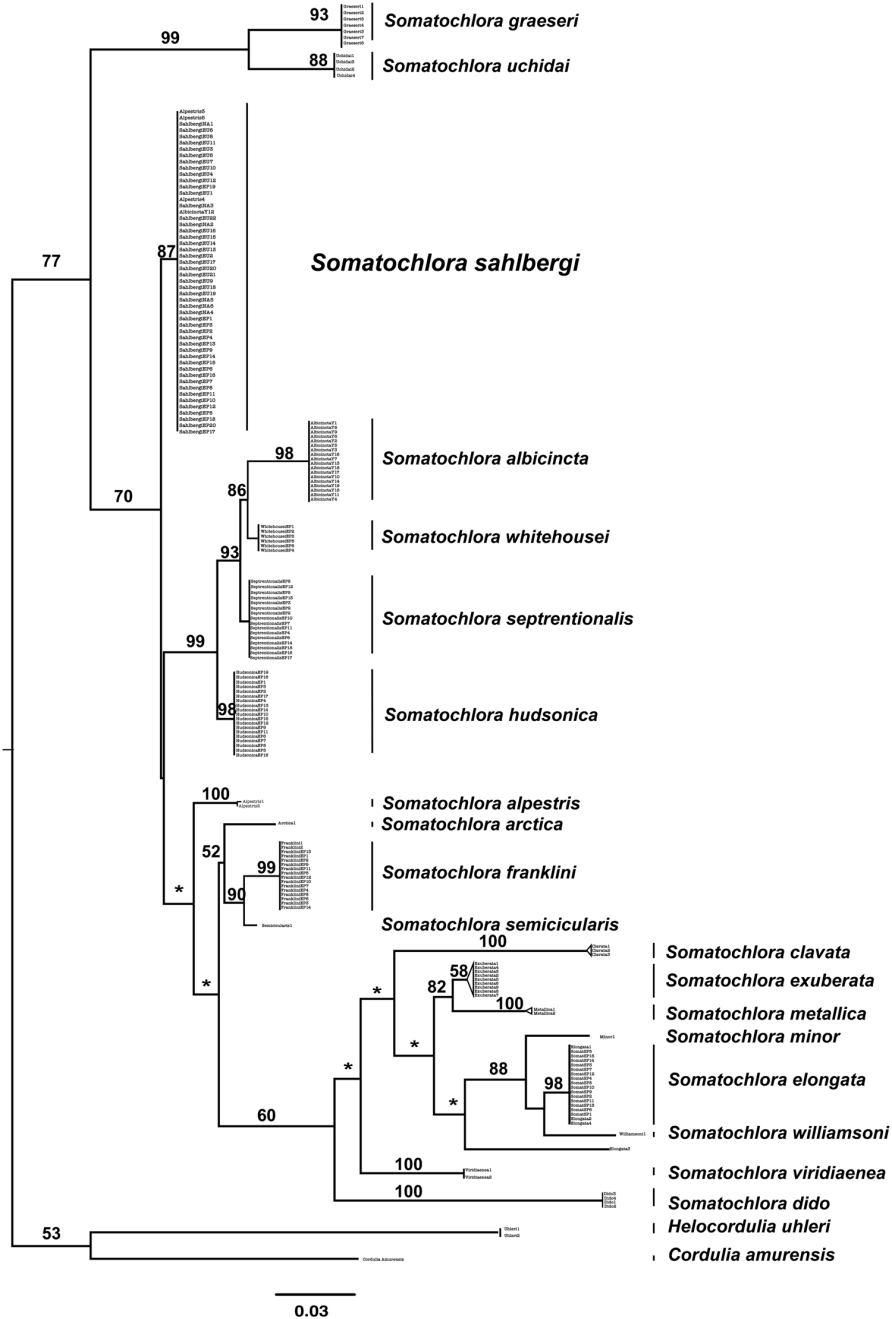
COI ML tree for genus *Somatochlora* — *S. sahlbergi* is recovered as a monophyletic group within *Somatochlora*. Numbers at the nodes represent the bootstrap values recovered using the ultrafast bootstrap method in IQTREE. For details on geographic sampling within *S. sahlbergi* see Fig. 3. Asterisks (“*”) on branches indicates bootstrap support lower than 50%.

To test the monophyly of *S. sahlbergi* we reconstructed a phylogenetic tree from COI dataset, comprising a total of 19 *Somatochlora* species, shown in Fig. 2. *Helocordulia uhleri* and *Cordulia amurensis* were used as outgroups. *Somatochlora graeseri* + *S. uchidai* form a clade (99% bootstrap support) that is sister to the remaining *Somatochlora*. At the next most apical split, *S. sahlbergi* is supported as a valid species with a bootstrap value of 87%. It is recovered as sister to the remaining species in *Somatochlora*, which form several multi-species clades.

*Somatochlora albicincta*, *S. whitehousei*, *S. septrentionalis*, and *S. hudsonica* are recovered in one clade with a bootstrap support of 99%. *Somatochlora minor*, *S. elongata* and *S. williamsoni* are recovered as a monophyletic clade with strong support (88%), as are *S. metallica* and *S. exuberata* (82% bootstrap support). *S. franklini* is also recovered as a monophyletic group with 99% support.

**Figure 3:**
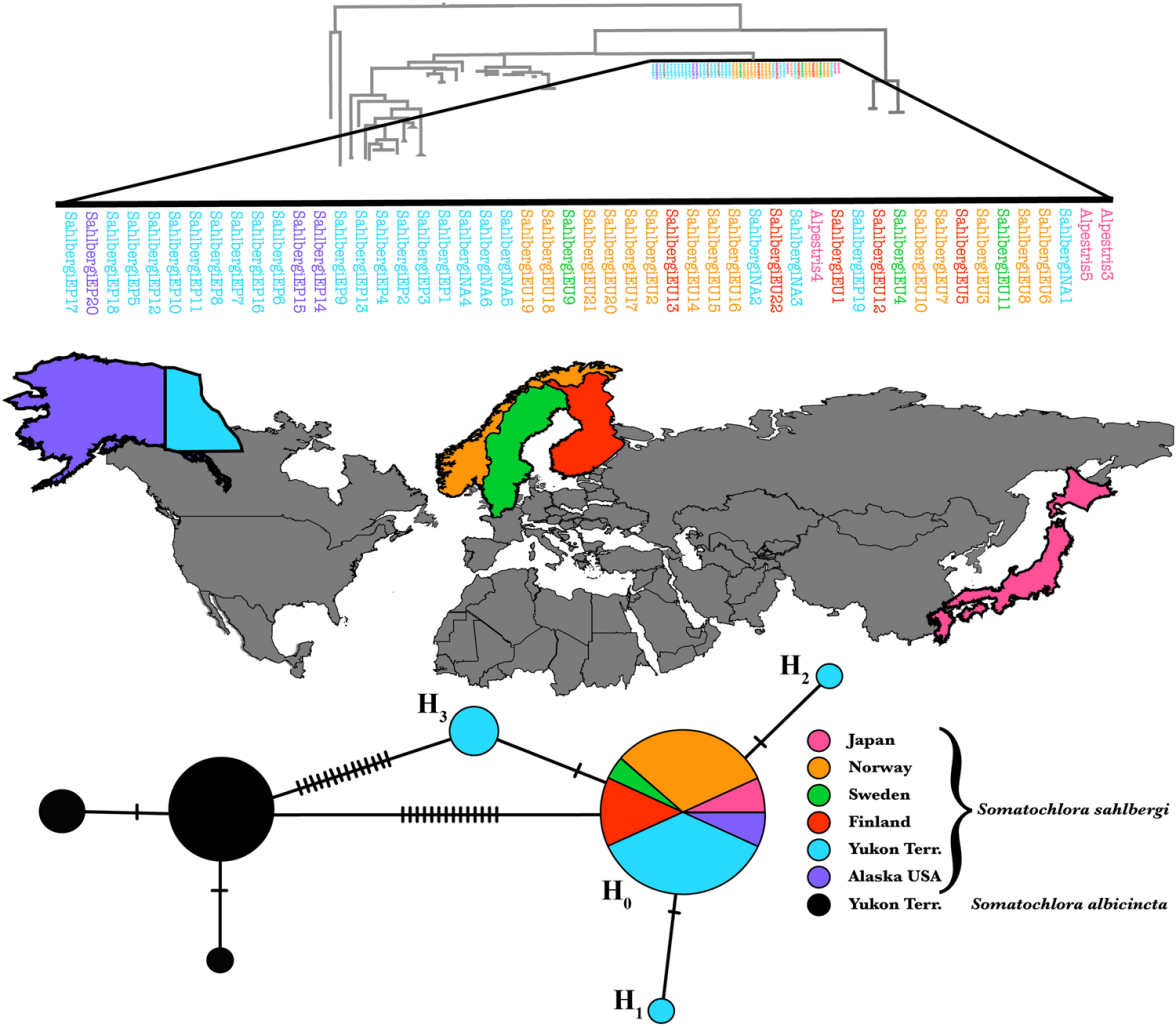
COI haplotype map of *S. sahlbergi* — different *S. sahlbergi* populations are colored according to their geographic location of origin. The haplotype map represents the relationship of all the populations of *S. sahlbergi*, distributed in four different haplotypes; H_0_, H_1_, H_2_ and H_3_ and *S. albicincta*. Within *S. sahlbergi*, 45 out of 51 samples share haplotype H_0_. The remaining 6 samples fall in 3 different haplotypes. The phylogenetic position of all the *S. sahlbergi* samples are color coded according to their geographic affinities.

Interestingly, three NCBI samples, identified by accession numbers AB708910.1, AB708909.1 and AB708908.1, are labelled as “*S. alpestris”* on GenBank, yet were consistently recovered within the monophyletic *S. sahlbergi* clade (Figs 2 and 3) in our analysis. These sequences originated from Karube *et al*.^32^, who published a total of five sequences from Hokkaido, Japan, each identified as *S. alpestris*. We included all 5 sequences^32^ in our tree reconstruction: three were recovered within the *S. sahlbergi* clade while the other two were recovered as a separate clade with 100% bootstrap support (Fig. 2).

For the CO1 + D2 dataset, we included *S. sahlbergi* and *S. albicincta* sequenced *de novo* from the Yukon and Europe. *Helocordulia uhleri* was used as the outgroup in this analysis. *Somatochlora albicincta* was recovered as a valid species with 86% bootstrap support, while *S. sahlbergi* was recovered as a valid species with 74% bootstrap support (Supp. Fig. S1). Within *S. sahlbergi*, samples were recovered in a polytomy with short branch lengths, suggesting that these sequences have only few nucleotide differences between them.

We conclude from our phylogenetic analysis that *S. sahlbergi*, sampled across its Holarctic range, comprises a single species from its well-supported monophyly in the tree; however, we hesitate to draw further conclusions regarding the phylogenetic relationships among other members of the genus *Somatochlora*. We recommend additional gene and taxon sampling among *Somatochlora* before drawing such conclusions.

### Haplotype analysis

Results from our haplotype analysis of *S. sahlbergi* using the gene COI are shown in Fig. 3. These results are congruent with the phylogenetic analysis, showing very little variation among populations of *S. sahlbergi*. The analysis included 51 samples collected from Sweden (3), Finland (5), Norway (14), the Yukon (23), Alaska (3) and Japan (3: GenBank samples that had been recovered as *S. sahlbergi* in our phylogenetic reconstruction). We recovered a total of four different haplotypes, labeled H_0_, H_1_, H_2_, and H_3_. Haplotype H_0_ is shared by 45 out of 51 samples that were used in the analysis. These 45 samples were collected across the three continents, which leads us to conclude that *S. sahlbergi* do not show any signature of genetic differentiation by distance. H_1_ and H_2_ each comprise a single sample collected in the Yukon Territory. H_3_ contains 4 samples collected in Yukon territory.  H_1_, H_2_, and H_3_ each differ from H_0_ by a single nucleotide difference.

### Intraspecific diversity

**Figure 4:**
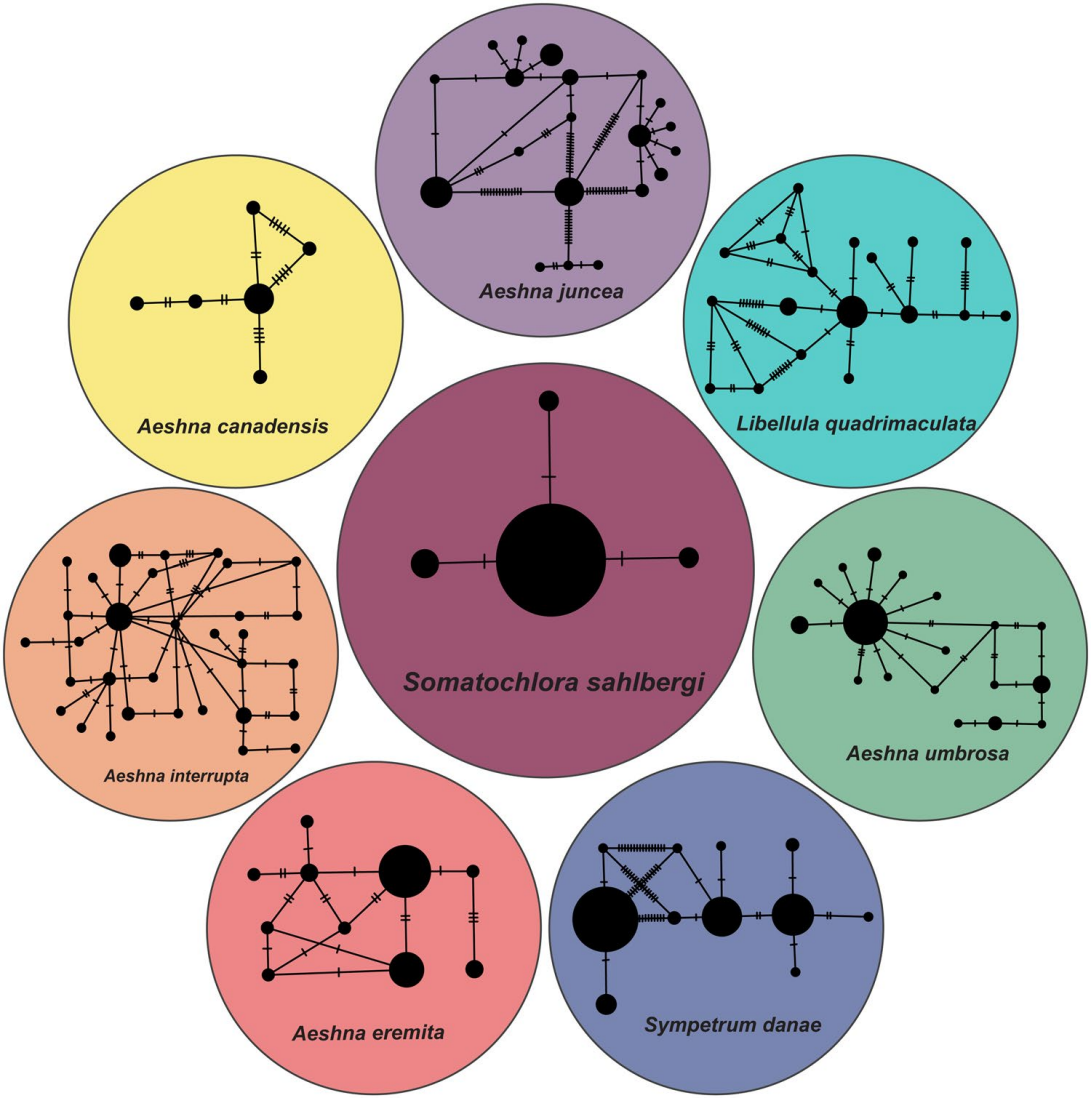
Comparative haplotype networks of northern odonates — seven northern species compared to *S. sahlbergi*, which shows less variation in its COI gene than do the other species. Circles represent haplotypes and slashes over a line connecting two haplotypes represent the number of nucleotide differences between them. See Supp. Tables S4 and S5 for more details.

Levels of intraspecific diversity were lowest in *S. sahlbergi* and *S. albicincta* (PID = 0.1%) among the 12 northern species (Fig. 4, Supp. Table S5). *Aeshna juncea* (PID = 1.9%), *A. subarctica* (PID = 2.2%)*, L. quadrimaculata* (PID = 1.0%) and *S. danae* (PID = 3.6%) each had almost tenfold higher intraspecific diversity levels than *S. sahlbergi. Aeshna umbrosa* showed 0.7% intraspecific divergence, *A. canadensis* and *A. interrupta* showed 0.5% divergence while *Leucorrhinia glacialis* and *A. eremita* showed 0.4% and 0.3% divergence respectively. *A. septentrionalis* showed the second lowest sequence divergence at 0.2%. We got the same values of intraspecific diversity using KP2 and JC nucleotide evolution models.

### Larval morphology

All but three *S. sahlbergi* larvae lacked lateral spines on segments 8 and 9 (with: N = 3, without: N = 15). This lack of spines did not appear to be associated with larval stadium.

## Discussion

Here, we present the first comprehensive phylogenetic reconstruction of *Somatochlora sahlbergi* and double its known localities from approximately 71^8^ to around 140 (Supp. Table S1). Our results support three main conclusions regarding *S. sahlbergi*: (1) it is a single species across its entire geographic range, with low levels of intraspecific genetic diversity; (2) the presence/absence of lateral spines is not a reliable character for distinguishing its larvae from those of other *Somatochlora* species; and (3) its range seems to include Japan.

### *Somatochlora sahlbergi* is a single species across its geographic range

Despite populations in North America and Europe being divided by oceans, our results suggest that *S. sahlbergi* populations may be panmictic across their Holarctic range. We recovered four different haplotypes among all the populations of *S. sahlbergi*, but most of the individuals, sampled from different continents, shared one single haplotype (Fig. 3). The additional haplotypes (H_1_, H_2_ and H_3_) differ from the main one (H_0_) by a single nucleotide difference each, but this does not indicate any significant difference within the populations because a one-nucleotide difference can be captured in a sequence just by chance (e.g., due to a sequencing error). Additionally, in comparison to other northern odonates, *S. sahlbergi* shows the lowest amount of intraspecific divergence (PID = 0.1%) in the COI. The only other species to show intraspecific sequence divergence of 0.1% is *S. albicincta*; however, this value can be expected in *S. albicincta* because all the samples used in the analysis were collected from a small geographic location. In fact, most of the northern species, despite having smaller geographic ranges, show more intraspecific variation across their populations in comparison to *S. sahlbergi* (Supp. Table S5).

Along with low intraspecific diversity, *S. sahlbergi* also shows low haplotypic diversity. Figure 4 shows haplotype networks for some northern species in comparison to *S. sahlbergi*. *Aeshna juncea*, for example, a circumboreal dragonfly that overlaps with *S. sahlbergi* in its northern range and exhibits a large haplotypic diversity, indicating much higher genetic variation than that seen in *S. sahlbergi* (sample size of *A. juncea*: 57; sample size of *S. sahlbergi*: 51). Similarly, *Libellula quadrimaculata*, which is found in both Europe and North America, also shows high haplotype diversity with a complex network connecting these haplotypes. We found that the D2 region of ribosomal 28S, known for being highly variable due to the presence of loops in its secondary structure, was extremely invariable in *S. sahlbergi*. Below we discuss four hypotheses that either alone or in a combination may explain the low genetic variation seen in the COI and D2 genes in *S. sahlbergi*.

First, gene flow might be prevalent among the North American and European populations. It would follow that individuals of *S. sahlbergi* are moving (likely through flight) between North America and Eurasia and mating. There are two possible directions of this movement: individuals may travel from western North America, through eastern North America (where they do not appear to live), and into Europe and Russia over the Atlantic Ocean; or they may fly the shorter distance over the Bering Sea (See Fig. 1). In either case, these insects would be dispersing for thousands of kilometers. *Anax junius* Drury^33^ and *A. ephippiger* (Burmeister)^34^ have been recorded travelling large distances and crossing vast water-bodies; however, the longest transoceanic migration ever recorded for a dragonfly (and any insect for that matter) is accomplished by *Pantala flavescens* (Fabricius). This dragonfly is capable of migrating a remarkable distance of 3,500 km across Indian Ocean^35,36^. Troast *et al*.^17^ concluded that *P. flavescens* is a panmictic species, with gene flow among its various populations across the globe. *S. sahlbergi* shows a similar trend, with little genetic variation across its global populations. However, there is a difference in the possible mode of dispersion for these dragonflies: *P. flavescens* is a passive flier, dependent on wind systems like intercontinental trade winds to carry it across oceans^17^, whereas *S. sahlbergi*, with its comparatively narrow wings would have to rely more on active flight, engaging in more flapping flight than gliding to stay airborne. There are examples of active flying, migratory dragonfly species (e.g., *A. junius*^37^); however, none of them are known to fly as far as *P. flavescens*, or over the distances that *S. sahlbergi* individuals would need to fly to maintain gene flow between populations. Based on a few observations of patrolling behaviour exhibited by males, *S. sahlbergi* are considered to be “flyers”, which spend the majority of their active time flying rather than perching^3,14,38^. Yet, due to the elusive nature of this species, there are no empirical records of the distance they are capable of travelling. Radio-transmitters have been successfully used to track dispersal distance and direction of *A. junius*^39^, but the smaller *S. sahlbergi* may not be able to support the weight of these devices. Alternatively, an isotope analysis^40^ of wings may help determine whether individuals are migrating across the Northern Atlantic Ocean or Bering Sea. To test this hypothesis, we are now extending our sampling to the Russian population.

Second, the time since divergence between the populations may be relatively short, indicating that populations *S. sahlbergi* were connected until relatively recently. Assuming this species had the same geographic range in its past as it does now, then during the last glaciation these populations would have been connected to each other through Beringia. The Beringian land bridge, which spanned the modern-day Bering Strait, disappeared relatively recently when the sea level rose around 11,500 cal BP^41^. Perhaps members of *S. sahlbergi* were able to maintain migration along that route for a while after the bridge disappeared. This hypothesis can be tested by estimating the divergence time between populations using coalescence models^42,43^. However, results from coalescence analysis using our current data set will not be reliable since the lack of genetic variation in our DNA sequences will lead to recovering artificially low substitution rates.

Third, long generation times in *S. sahlbergi* may explain their lack of genetic diversity. Molecular evolution rates in species are thought to be correlated with generation time: the smaller the generation time, the faster the rate of molecular evolution. This correlation has been found in several groups of organisms, including invertebrates^44^. In temperate regions, odonate life cycles become increasingly long with increasing latitude as larval growth can be arrested by a reduction in photoperiod and temperature^6^. Individuals of *S. sahlbergi* survive in places where the water is frozen for at least eight months out of the year (observations by GS). Therefore, perhaps this species takes several years to complete its life cycle. Ulf Norling studied life cycle length in two northern odonate species, *Leucorrhinia dubia* (Vander Linden) and *Coenagrion hastulatum* Charpentier, at 67°N. He found that *L. dubia* had an approximately 4-year development period^45^ while *C. hastulatum* took 3–4 years to develop^46^. Based on these observations, it is reasonable to assume that the very short summers in the north add at least one extra year to the life cycle for *S. sahlbergi*, suggesting 4–5 years of total larval development time, which is supported by analyses of various larval instars by Cannings and Cannings^3^. Thus, a relatively long generation time, combined with a relatively short time since divergence (as discussed above) might have led to very low levels of genetic variation in *S. sahlbergi*.

Fourth and finally, *S. sahlbergi* may have suffered a genetic bottleneck at some point in its evolutionary history. Genetic bottlenecks, resulting in a dramatic decrease in genetic diversity, can be caused by events such as the introduction of new pathogens or predators, or habitat loss (perhaps caused by a change in climate). Due to their proximity to glacial ice sheets, populations of *S. sahlbergi* have likely experienced many climatic fluctuations in their evolutionary past. During the last glacial termination (17.7–11.5 kBP), the Northern hemisphere saw periods of cold/warm oscillations as the glaciers were receding^47^. This was marked by an intense period (Younger Dryas: 12.9-11.7 kBP) marked by a cold and dry climate^48^. The relatively stable Holocene climate has also seen several intervals of rapid climate change (e.g., the Little Ice Age and Medieval Warm Period) that affected the Northern Hemisphere^49^. These climatic fluctuations might have affected some populations of *S. sahlbergi* more severely than other, perhaps more southern populations, especially in North America and Europe, leading to their local extinction. Additionally, the Holocene fluctuations after the last glaciation may have made recolonization difficult for the few remaining individuals.

### Larval morphology

#### Lateral spines

Larvae of *S. sahlbergi* are described as having dense and fairly-long setae covering their abdomens, with prominent lateral spines on segments 8 and 9^2^; however, we found that these characteristics were not always present, especially in the case of lateral spines. Out of the total 24 larvae from northern Europe that genetically group as *S. sahlbergi*, only 3 had lateral spines. This is in contrast to samples from Norway examined previously by Norling and Sahlén^4^, as well as the description of the larva from Canada^3^ where all larvae possessed lateral spines. Absence of these lateral spines in larvae makes *S. sahlbergi* difficult to distinguish from other sympatric *Somatochlora* species (*S. albicincta* in the Yukon and *S. alpestris* in Europe, which sometimes occupy similar habitats).

#### Color

**Figure 5:**
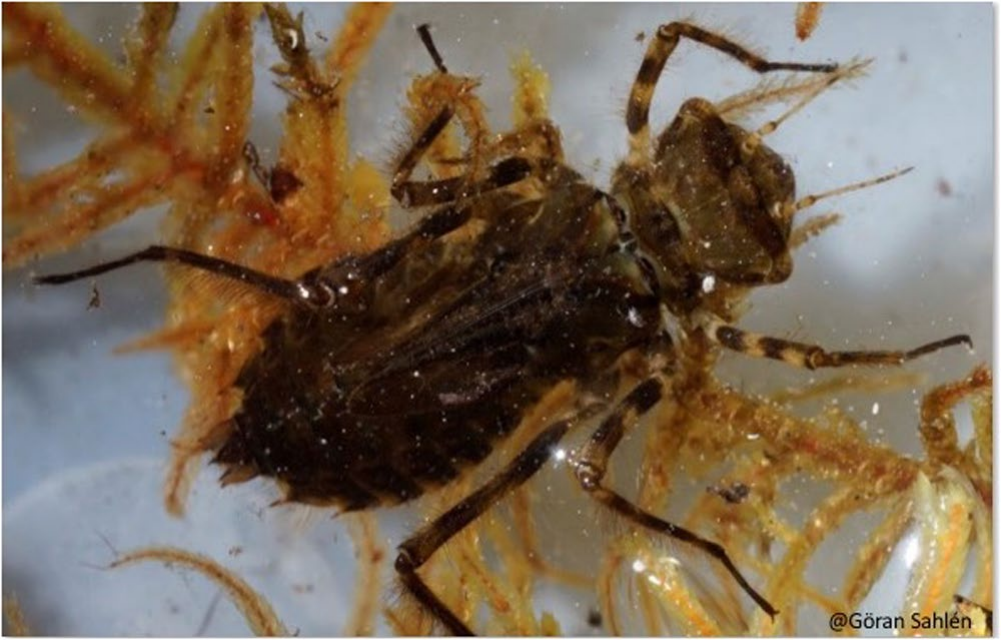
Larva of *S. sahlbergi* — notice the dark brown coloration on the dorsum of the larva (collected in Finland). Photo credit: GS.

All the 24 larval specimen that were collected by GS and JLW in Europe were dark brown in color (Fig. 5), unlike the orange-brown suggested by Cannings and Cannings^3^. The underside of these larvae were greyish/green-brown and we did not see any specimen with a yellow underside. We suspect that the reddish/orange color observed by both Cannings and Cannings^3^ and Valle^50^ might be an artefact of the preservation method rather than the original color of a freshly caught larvae. Mud or mineral deposits (for example iron) can cause larvae to appear a different color than the original.

### Japan: A new location for *Somatochlorasahlbergi*

*S. sahlbergi* was described from a sample collected in Siberia^51^. Since then, it has been recorded around the Arctic and subarctic regions (Fig. 1). Based on our results, we propose that this species may also be present in Japan. *S. sahlbergi* has not been recorded in Japan before, but has been found in the Kamchatka peninsula in Russia, which is ~2,700 km from Japan. Three samples that have been identified as *S. alpestris* on GenBank can either be a case of misidentification or may demonstrate hybridization between *S. sahlbergi* and *S. alpestris*. *S. sahlbergi* has been known to hybridize with other congeneric *Somatochlora* species like *S. hudsonica* and *S. albicincta*^3,7^. Therefore, it is important to further study the *Somatochlora* that are found in northern Japan to gain further insight into the history of this group and of *S. sahlbergi*.

### Habitat notes

Based on previous literature, *S. sahlbergi* has been considered a habitat specialist^5,8^, often recorded in or near a rather specialized Arctic habitat called a “palsa mire”; however, our recent observations in northwestern Canada, Sweden, Norway and Finland and records from Kamchatka indicate that *S. sahlbergi* may not be restricted to palsa mires^7,52^. We suggest instead that this species inhabits a variety of habitat types.

In Sweden, Norway and Finland, larvae were taken from small to large bodies of slowly-seeping water, which were covered in mosses and directly connected to deep, cold springs (*pers. obs*. by GW and JLW). In northwestern Canada, *S. sahlbergi* has been associated with pools and small lakes that have “aquatic moss as the dominant vegetation and deep, cold water”^3^.

In August 2015, MKK and WRK surveyed 70 distinct sites in the Yukon and Northwest Territories along the Dempster Highway between the Ogilvie Mountains to the south and the Mackenzie River to the northeast; these included eight localities in the Yukon Territory where *S. sahlbergi* had previously been spotted or collected between 1980 and 2010 (SG Cannings, *pers. comm*.). *S. sahlbergi* was found in only two of these localities, and only one those two was previously recorded by Cannings. The first *S. sahlbergi* site (Supp. Table S1, site code CA150812-03) was a pond, surrounded by tall trees, shrubs and sedges, with moss on its bottom. The water was 39–54 cm deep and approximately 14 °C. A single *S. sahlbergi* larva was collected along with several aeshnid larvae. The second site (site code CA150815-05) was a pair of connected ponds, with a shallow region of sedges between them, surrounded by trees. Three male and one female *S. sahlbergi* adults were collected, primarily flying over this sedge area, as well as one *S. albicincta* larva. It is interesting, and perhaps alarming, to note that *S. sahlbergi* had apparently disappeared (or at least were undetected) from seven of Cannings’ previous sites.

Given this evidence, we conclude that *S. sahlbergi* is perhaps not as much of a specialist as previously suggested^5,8^ in the literature. It is important to come to a consensus on the habitat of this species because we may not have the opportunity to study this system for long, as *S. sahlbergi* inhabits the edge of a climatic zone that is at immediate risk from climate change. Observations from a recent expedition to the Yukon (by MKK and WRK) and recent studies in the Scandinavian region^5^ suggest that *S. sahlbergi* might already be facing habitat loss in these locations because of temperature increase caused by global climate change^53,54^. Additionally, there is evidence that *S. sahlbergi* is facing competition from *S. metallica* in Eurasia, which is moving north due to global warming^5^. This study is a first step toward exploring the evolutionary and biogeographical history of *S. sahlbergi*, a single species that shows surprisingly low genetic diversity in its global distribution and is likely under stress from warming and competition.

## Conclusion

*S. sahlbergi* is a single, panmictic species across the North American and European section of its range. Populations of *S. sahlbergi* show very little genetic variation in comparison to other dragonfly species from northern latitudes. It is presently unclear whether *S. sahlbergi* individuals are dispersing long distances and if so, whether they cross the ocean. We also conclude that lateral spines are not an identification character for *S. sahlbergi* compared to other congenerics. Future work should examine material from the Asian part of the geographic range of this remarkable Arctic dragonfly.

## Electronic supplementary material

Supplementary Material

## Data Availability

Sequences used in the study are available on GenBank (see Supp. Table S2 for accession numbers). Alignments created during the analysis are available on request from the corresponding author.
