# Supplementary material for: Extremely low genetic diversity in a circumpolar dragonfly species, Somatochlora sahlbergi (Insecta: Odonata: Anisoptera)
Source: Sci Rep. 2018 Oct 11;8:15114. doi: 10.1038/s41598-018-32365-7 (PMC6181962; doi:10.1038/s41598-018-32365-7)
Supplement: Supplementary file 1 — Supplementary Material [file 41598_2018_32365_MOESM1_ESM.pdf]

Extremely low genetic diversity in circumpolar dragonfly species, *Somatochlora sahlbergi* (Insecta: Odonata: Anisoptera)

Manpreet K. Kohli, Göran Sahlén, William R. Kuhn, Jessica L. Ware

Supplementary Information

Table of Contents

Supplementary Table S1. List of known localities for *Somatochlora sahlbergi* 2
Supplementary Table S2. List of specimens used in phylogenetic reconstruction of *Somatochlora* 11
Supplementary Table S3. List of primers 17
Supplementary Table S4. List of specimens used in haplotype networks 18
Supplementary Table S5. Intraspecific variation in 12 Holarctic dragonfly species 22

Supplementary Figure S1. COI+D2 Tree 23

References 24

Supplementary Table S1. List of known localities for *Somatochlora sahlbergi*. Records are sorted by longitude, latitude, and date.

Coordinates (in dec. deg.)	Coord. Type[a]	Elevation	Record Date (M/D/Y)	Country	State /Province	Locality Description[b]	Collector/Observer | Deposition Information[c]	Source[d]
43.770108, 142.918046	X		8/11/1998	Japan	Hokkaido	Kamikawa	Takahiko Yagi | GenBank accession nos. AB708909.1 (COI), AB707965.1 (16S), AB707015.1 (18S, ITS1, 5.8S, ITS2, 28S); labeled as S. alpestris	FUTashi 2012 via GenBank
43.770108, 142.918046	X		8/17/2000	Japan	Hokkaido	Kamikawa	GenBank accession nos. AB708910.1 (COI), AB707966.1 (16S), AB707016.1 (18S, ITS1, 5.8S, ITS2, 28S); labeled as S. alpestris	FUTashi 2012 via GenBank
43.770108, 142.918046	X		8/30/2009	Japan	Hokkaido	Kamikawa	GenBank accession nos. AB708908.1 (COI), AB707964.1 (16S), AB707014.1 (18S, ITS1, 5.8S, ITS2, 28S); labeled as S. alpestris	FUTashi 2012 via GenBank
49.361219, 110.187381	E		≤2011	Russia	Zabaykalsky Krai	southern Siberia		Schröter 2011, est. Fig. 4
49.83444, 84.34101	E		1973	Kazakhstan	Zyryan District	east of Irtysh River toward Kazakhstan-Russia border		Belyshev 1973, est. Fig. 154
50.331428, 87.750424	E		≤2011	Russia	Altai Republic	southern Siberia, South-eastern Altaj Mts. – Kuranskyi mountain range – north-eastern from Atkash village [Юго-Восточный Алтай – Курайский хребет – село Акташ]		Kosterin 1989, via Holuša 2009
51.466667, 156.9, coords. labeled as “circa”	E	81 m	6/25/1978	Russia	Kamchatka Krai	Oxbows of Pauzhetka River at Pauzhetka village	A Haritonov	Dumont et al. 2005, p. 46, “location 57”
51.466667, 156.9, coords. labeled as “circa”	E	81 m	6/25/1978	Russia	Kamchatka Krai	Oxbows of Pauzhetka River at Pauzhetka village	A Haritonov	Dumont et al. 2005, p. 46, “location 57”
51.466667, 156.9, coords. labeled as “circa”	E	81 m	7/11/1978	Russia	Kamchatka Krai	Oxbows of Pauzhetka River at Pauzhetka village	A Haritonov	Dumont et al. 2005, p. 46, “location 57”
51.466667, 156.9, coords. labeled as “circa”	E	81 m	7/11/1978	Russia	Kamchatka Krai	Oxbows of Pauzhetka River at Pauzhetka village	A Haritonov	Dumont et al. 2005, p. 46, “location 57”
51.466667, 156.9, coords. labeled as “circa”	E	81 m	7/12/1978	Russia	Kamchatka Krai	Oxbows of Pauzhetka River at Pauzhetka village	A Haritonov	Dumont et al. 2005, p. 46, “location 57”
51.466667, 156.9, coords. labeled as “circa”	E	81 m	7/12/1978	Russia	Kamchatka Krai	Oxbows of Pauzhetka River at Pauzhetka village	A Haritonov	Dumont et al. 2005, p. 46, “location 57”
51.542634, 133.835633	E		≤2011	Russia	Khabarovsk Krai	eastern Siberia		Schröter 2011
51.54271, 118.2819	E		1973	Russia	Zabaykalsky Krai	between Ergun and Shilka Rivers, W of Russia-China border		Belyshev 1973, est. Fig. 153
51.7368, 102.83821	E		1973	Russia	Buryatia	Tunkisnsky District, near Irkut and Zun-Muren Rivers		Belyshev 1973, est. Fig. 151
51.738534, 102.545193	E		1961	Russia	Buryatia	southern Siberia, Tunkinskaja valley near Turan village [Тункинская котловина – близ села Туран]		Belyshev & Ovodov 1961, via Holuša 2009
52.01511, 113.395	E		1973	Russia	Zabaykalsky Krai	Ingoda River near Kenon Lake		Belyshev 1973, est. Fig. 152
52.2549, 104.2273	E		1973	Russia	Irkutsk Oblast	Confluence of Irkut and Angara Rivers near Irkutsk		Belyshev 1973, est. Fig. 143
52.45099, 85.03418	E		1973	Russia	Altai Krai	Smolensky District, where Biya and Katun Rivers meet to form Ob River		Belyshev 1973, est. Fig. 133

Coordinates (in dec. deg.)	Coord. Type[a]	Elevation	Record Date (M/D/Y)	Country	State /Province	Locality Description[b]	Collector/Observer | Deposition Information[c]	Source[d]
52.616216, 90.07993	E			Russia	Republic of Khakassia	southern Siberia, West Sajan Mts. surrounding Abaza village		Kharitonov 1990, via Holuša 2009
52.66972, 76.72302	E		1973	Kazakhstan	Pavlodar District	Irtysh River near Pavlodar		Belyshev 1973, est. Fig. 134
53.089444, 157.878611	E		2000	Russia	Kamchatka Krai	Mesotropic lake		Kalkman & Dijkstra 2000
53.089444, 157.878611	E		2000	Russia	Kamchatka Krai	Mesotropic lake		Kalkman & Dijkstra 2000
53.089444, 157.878611	X	500 m	7/23/2003	Russia	Kamchatka Krai	medium-sized mesotrophic lake with a smaller round lake nearby, situated at foot of Vackazhets Mt.		Dumont et al. 2005, p. 46, "location 33"
53.089444, 157.878611	X	500 m	7/23/2003	Russia	Kamchatka Krai	medium-sized mesotrophic lake with a smaller round lake nearby, situated at foot of Vackazhets Mt.		Dumont et al. 2005, p. 46, "location 33"
53.089444, 157.878611	X	500 m	7/24/2003	Russia	Kamchatka Krai	medium-sized mesotrophic lake with a smaller round lake nearby, situated at foot of Vackazhets Mt.		Dumont et al. 2005, p. 46, "location 33"
53.089444, 157.878611	X	500 m	7/24/2003	Russia	Kamchatka Krai	smaller round lake near medium-sized lake, situated at foot of Vackazhets Mt.		Dumont et al. 2005, p. 46, "location 33"
53.089444, 157.878611	X	500 m	7/24/2003	Russia	Kamchatka Krai	medium-sized mesotrophic lake with a smaller round lake nearby, situated at foot of Vackazhets Mt.		Dumont et al. 2005, p. 46, "location 33"
53.089444, 157.878611	X	500 m	7/24/2003	Russia	Kamchatka Krai	smaller round lake near medium-sized lake, situated at foot of Vackazhets Mt.		Dumont et al. 2005, p. 46, "location 33"
53.42099, 83.79272	E		1973	Russia	Altai Krai	Ob River near Barnaul		Belyshev 1973, est. Fig. 132
53.80227, 91.49963	E		1973	Russia	Krasnoyarsk Krai	Yenisei River near Minusinsk in Kras. Krai and Abakan in Republic of Khakassia		Belyshev 1973, est. Fig. 140
54.1037, 67.73895	E		1973	Kazakhstan	Shal akyn District	Ishim River SW of Ornek		Belyshev 1973, est. Fig. 138
54.942211, 83.066857	E		≤2011	Russia	Novosibirskaya Oblast			Schröter 2011, est. Fig. 4
54.97919, 83.02094	E		1973	Russia	Novosibirsk Oblast	Novosibirsk, Ob River near confluence with Inya River		Belyshev 1973, est. Fig. 131
56.22198, 93.38928	E		1973	Russia	Krasnoyarsk Krai	Yemelyanovsky District, Yenisei River NE of Krasnoyarsk		Belyshev 1973, est. Fig. 139
56.29931, 90.49441	E		1973	Russia	Krasnoyarsk Krai	Chulym River near Achinsk		Belyshev 1973, est. Fig. 141
56.60033, 66.2915	E		1973	Russia	Tyumen Oblast	Yarkovsky District, confluence of Iset and Tobol Rivers		Belyshev 1973, est. Fig. 138
56.8775, 84.53979	E		1973	Russia	Tomsk Oblast	Tomsky District, confluence of Ob and Tom Rivers		Belyshev 1973, est. Fig. 129
57.21942, 66.94021	E		1973	Russia	Tyumen Oblast	Yarkovsky District, confluence of Tura and Tobol Rivers		Belyshev 1973, est. Fig. 137
58.16781, 68.24158	E		1973	Russia	Tyumen Oblast	confluence of Tobal into Irtysh River near Tobolsk		Belyshev 1973, est. Fig. 135
58.36805, 82.64008	E		1973	Russia	Tomsk Oblast	Kolpashevsky Disctrict, confluence Ket and Ob Rivers		Belyshev 1973, est. Fig. 128

Coordinates (in dec. deg.)	Coord. Type[a]	Elevation	Record Date (M/D/Y)	Country	State /Province	Locality Description[b]	Collector/Observer | Deposition Information[c]	Source[d]
59.000114, 126.213686	X	~280 m	6/24/2002	Russia	Sakha Republic	Aldan River left bank 7-9 km upstream of Tommot town		Kosterin 2004
59.00238, 91.67816	E		1973	Russia	Krasnoyarsk Krai	Yeniseysky District, Yenisei River at confluence of Bolshoy Pit River		Belyshev 1973, est. Fig. 142
59.75, 163.333333	X		7/24/1994	Russia	Kamchatka Krai	Tovuvnan Mountiain foot		Dumont et al. 2005, p. 46, “location 2”
59.75, 163.333333	X		7/24/1994	Russia	Kamchatka Krai	Tovuvnan Mountiain foot		Dumont et al. 2005, p. 46, “location 2”
60.1415, 78.96973	E		1973	Russia	Tomsk Oblast	Alexandrovsky District, Ob River		Belyshev 1973, est. Fig. 130
60.86, -161.41	E		<1917	USA	AK	Kuskokwim River	A Stecker | CM	SGC
62.06273, 129.7925	E		1973	Russia	Sakha Republic	Lena River near Yakutsk		Belyshev 1973, est. Fig. 144
62.445024, 152.234298	E		≤2011	Russia	Magadan Oblast	eastern Siberia		Schröter 2011
62.45395, 88.94531	E		1973	Russia	Krasnoyarsk Krai	confluence of Bachta River with Yenisei River		Belyshev 1973, est. Fig. 147
63.6617, -144.0737	X		7/17/1987	USA	AK	Dot Lake, beside AK HWY, pond	SG Cannings | SEM 5279	SGC
63.781, -145.7505	X		7/19/1895	USA	AK	Donnelly Dome, 20 mi S of Delta Junction on Richardson HWY, rocky-bottomed pond in willow/alder tundra	SG Cannings | SEM 5275–5277	SGC
63.781, -145.7505	X		7/15/1985	USA	AK	Delta Junction, 20 mi S on Richardson HWY, sedge-bordered, rocky-bottomed pond	SG Cannings | SEM 5272, 5273	SGC
63.781, -145.7505	X		7/20/1985	USA	AK	Donnelly Dome, 20 mi S of Delta Junction on Richardson HWY	SG Cannings | SEM 2225, 2226	SGC
63.781, -145.7505	X		7/15/1987	USA	AK	Donnelly Dome, 20 mi S of Delta Junction on Richardson HWY, moss & rocky-bottomed pond	SG Cannings | SEM 2222, 5278	SGC
63.781, -145.7505	X		7/17/1987	USA	AK	Donnelly Dome, 20 mi S of Delta Junction on Richardson HWY, pond	SG Cannings | SEM 2223, 2224	SGC
63.781, -145.7505	X		6/17/2010	USA	AK	Donnelly Dome, treeline pond 2 km E (Delta Junction, 28.5 km S)	E Neipert | DTA	SGC
63.781, -145.7505	X		7/7/2010	USA	AK	Donnelly Dome, treeline pond 2 km E (Delta Junction, 28.5 km S)	E Neipert | RBCM	SGC
63.781, -145.7505	X		7/8/2010	USA	AK	Donnelly Dome, treeline pond 2 km E (Delta Junction, 28.5 km S)	E Neipert | DTA	SGC
65.0388, -138.16977	X		7/26/2010	Canada	YT	Blackstone River, km 141 Dempster HWY	SG Cannings | RBCM	SGC
65.0635, -138.13	X		6/30/1982	Canada	YT	Blackstone River, km 148 Dempster HWY (old road km 145.5), deep Sphagnum bog pool rimmed with Carex	SG Cannings | SEM 2230, 5294–5296	SGC
65.0635, -138.13	X		6/25/1987	Canada	YT	Blackstone River, fend pond at km 148 Dempster HWY (old road km 145.5)	SG Cannings | SEM 2232	SGC
65.0635, -138.13	X		7/3/1995	Canada	YT	Blackstone River, km 150 Dempster HWY (probably old road km 145.5)	RCH Cannings | RBCM	SGC
65.0635, -138.13	X		7/17/2005	Canada	YT	Blackstone River, km 145.5 Dempster HWY	GE Hutchings | RBCM	SGC

Coordinates (in dec. deg.)	Coord. Type[a]	Elevation	Record Date (M/D/Y)	Country	State /Province	Locality Description[b]	Collector/Observer | Deposition Information[c]	Source[d]
65.0638, -138.1279	X		7/27/2009	Canada	YT	Blackstone River, km 145.5 Dempster HWY	SG & RA Cannings | RBCM	SGC
65.064083, -138.128017 ±3 m	X	842±3 m	8/15/2015	Canada	YT	Dempster HWY near km 145 marker, W side of road (opposite Blackstone River on E side of road), small & large pond with sedgy vegetated area in between (#CA150815-05)	MK Kohli & WR Kuhn | JLW	This study
65.1627, -147.325 ±50 m	X		6/27/2013	USA	AK	Steese HWY, Chatanika River, oxbow slough	John Hudson | UAM100337102, 100337103	SGC
65.2069, -138.324872	X		6/30/1982	Canada	YT	Engineer Creek, km 175.5 Dempster HWY, roadside bog	SG Cannings | SEM 5297	SGC
65.2069, -138.3249	X		6/30/1982	Canada	YT	Engineer Creek, km 175.5 Dempster HWY	SG Cannings | RBCM	SGC
65.2069, -138.3249	X		7/21/2009	Canada	YT	Engineer Creek, km 174.1 Dempster HWY (old road km 175.5)	SG & RA Cannings | RBCM	SGC
65.2069, -138.3249	X		7/25/2009	Canada	YT	Engineer Creek, km 174.1 Dempster HWY (old road 175.5)	SG & RA Cannings | RBCM	SGC
65.2613, -138.261392	X		6/30/1982	Canada	YT	Engineer Creek, km 182 Dempster HWY	RA Moore & SG Cannings | SEM 5298–5300, RBCM	SGC
65.4272, -138.2267	X		7/21/2009	Canada	YT	Ogilvie River, km 207 Dempster HWY	SG & RA Cannings | RBCM	SGC
65.4906, -138.229	X		8/10/1980	Canada	YT	Ogilvie River, km 215 Dempster HWY	RJ Cannings | SEM 5308	SGC
65.4988, -138.2318	X		7/20/1980	Canada	YT	Ogilvie River, km 216 Dempster HWY	ROM field party | ROM	SGC
65.5446, -138.2228	X		8/10/1980	Canada	YT	Ogilvie River, km 228 Dempster HWY	RJ Cannings | RBCM	SGC
65.6413, -138.1404	X		7/24/2009	Canada	YT	Ogilvie River, km 233.2 Dempster HWY	SG & RA Cannings | RBCM	SGC
65.6986, -138.076762	X		7/22/1980	Canada	YT	Ogilvie River, km 243 Dempster HWY	RJ Cannings | SEM 5292	SGC
65.702233, -138.066933 ±3 m	X	494±3 m	8/12/2015	Canada	YT	Dempster HWY, pond just off E side of road (#CA150812-03)	MK Kohli & WR Kuhn | JLW S66	This study
66.446, -136.697	X		7/3/1982	Canada	YT	Eagle River, Dempster HWY	DM Wood | SEM 7368, 7369, 7370	SGC
66.647053, 66.388406	E		1975	Russia	Yamalo-Nenets Autonomous Okrug	Siberia, Polar Ural, Labytnangi settlement (Приполярный Урал – окрестности Лабытнанги)		Kharitonov 1975, via Holuša 2009
66.758174, 67.902238	E		≤2011	Russia	Yamalo-Nenets Autonomous Okrug			Schröter 2011, est. Fig. 4
66.933, -136.32338	X	2000 ft	6/30/1979	Canada	YT	Richardson Mts, W slope, km 448.5 Dempster HWY (old road km 443), bog pond	SG Cannings | SEM 2227	
66.933, -136.32338	X		7/18/1980	Canada	YT	Richardson Mts, km 450 Dempster HWY (#800105)	ROM field party | SEM 2228, 2229	SGC
66.933, -136.32338	X		7/3/1982	Canada	YT	Richardson Mts, km 450 Dempster HWY	SG Cannings | SEM 5301, 7371–7380	SGC
66.933, -136.32338	X		7/3/1982	Canada	YT	Richardson Mts, km 450 Dempster HWY, 3 km N of "Cornwall Creek", W slope	SG Cannings & RA Moore | SEM 5302–5305	SGC
66.933, -136.32338	X		7/6/1982	Canada	YT	Richardson Mts, km 450 Dempster HWY, 3 km N of "Cornwall Creek", W slope	SG Cannings & RA Moore | SEM 5306, 5307	SGC

Coordinates (in dec. deg.)	Coord. Type[a]	Elevation	Record Date (M/D/Y)	Country	State /Province	Locality Description[b]	Collector/Observer | Deposition Information[c]	Source[d]
66.933, -136.32338	X		7/23/2009	Canada	YT	Richardson Mts, km 448.5 Dempster HWY (old road km 450)	SG & RA Cannings | RBCM	SGC
67.023694, 69.616006	E		≤2011	Russia	Yamalo-Nenets Autonomous Okrug			Schröter 2011, est. Fig. 4
67.361425, 32.991477	E		≤2011	Russia	Murmansk Oblast	Gorad Apatity		Schröter 2011, estimated from Fig. 3
67.42, -140.0937	X		7/5/1983	Canada	YT	Old Crow, 20 km SW, fen pond	RA Cannings | SEM 5311, 5312–5315, RBCM	SGC
67.5495, -139.9186	X		7/3/1983	Canada	YT	Porcupine River, 4 km SW of Old Crow, deep bog pond	SG Cannings | SEM 5311	SGC
67.5691, -139.678501	X		7/17/1981	Canada	YT	Old Crow, 6 km E, Klokut Camp	SG Cannings | SEM 5293	SGC
67.5734, -139.8234	X		7/2/1983	Canada	YT	Old Crow, SE edge of airstrip, Carex pond	RA Cannings | SEM 5309	SGC
67.5734, -139.8234	X		7/3/1983	Canada	YT	Old Crow, Carex marsh	SG Cannings | SEM 5310	SGC
67.5734, -139.8234	X		7/6/1983	Canada	YT	Old Crow, SE edge of airstrip, Carex pond	RA Cannings | SEM 5316	SGC
67.5734, -139.8234	X		7/7/1983	Canada	YT	Old Crow, SE edge of airstrip, Carex pond	RA & RJ Cannings | SEM 5317–5322	SGC
67.5734, -139.8234	X		7/10/1983	Canada	YT	Old Crow, SE edge of airstrip, Carex pond	RJ Cannings | SEM 5323–5325	SGC
67.5734, -139.8234	X		7/24/1984	Canada	YT	Old Crow, SE airport ponds	SG Cannings | SEM 5326	SGC
67.5767, -139.8297	X		7/26/1984	Canada	YT	Old Crow, cemetery fen	SG Cannings | SEM 2231	SGC
67.766667, 72.15	X			Russia	Yamalo-Nenets Autonomous Okrug			Степанов 2016
67.859944, 19.452191 ±100 m	X		7/5/2014	Sweden	Norrbottens	Akkar	Björn Anderson | ART 50842855	GBIF
67.859964, 19.452029 ±25 m	X		7/5/2014	Sweden	Norrbottens	Akkar	Björn Anderson | ART 44959325	GBIF
67.86778, 86.63544	E		1973	Russia	Krasnoyarsk Krai	Yenisei River		Belyshev 1973, est. Fig. 150
68.083333, 86.422222	E		7/23/1876	Russia	Krasnoyarsk Krai	Siberia, Plachino, along Yenisei River		Hämäläinen 2015, pp. 26-27
68.18381, 145.2832	E		1973	Russia	Sakha Republic	Ularovskaya River near Druzhina		Belyshev 1973, est. Fig. 146
68.22721, 87.42964	E		1973	Russia	Krasnoyarsk Krai	near Khantayskoye Reservoir		Belyshev 1973, est. Fig. 149
68.416667, 86.333233	E		7/19/1876	Russia	Krasnoyarsk Krai	Siberia, Chantaika (Chantajskoje), along Yenisei River		Hämäläinen 2015, pp. 26-29
68.448036, 21.049288 ±2500 m	X		8/1/2010	Sweden	Norrbottens	myrgölsområde NV Pulsujärvi	Claes Kyrk | ART 41789601	GBIF
68.4522, 21.026317	X		2014	Sweden	Norrbottens	E Dávvajávri	Jessica Ware, Göran Sahlén	This study
68.45349, 21.040468	X		2014	Sweden	Norrbottens	E Dávvajávri	Jessica Ware, Göran Sahlén	This study
68.457073, 21.019086 ±250 m	X		7/26/2010	Sweden	Norrbottens	Damm W f. Pulsujärvi	Hans A. Olsvik | ART 41766854, 41766855	GBIF
68.457891, 21.015931	X		2010	Sweden	Norrbottens	E Dávvajávri	Magnus Billqvist	Magnus Billqvist, pers. comm.

Coordinates (in dec. deg.)	Coord. Type[a]	Elevation	Record Date (M/D/Y)	Country	State /Province	Locality Description[b]	Collector/Observer | Deposition Information[c]	Source[d]
68.459579, 21.000024 ±278m	X		7/22/2016	Sweden	Norrbottens	Småsjö med kallkällor, V Pulsujärvi	Magnus Billqvist, Ola Elleström | ART 100827799	GBIF
68.460918, 20.973479 ±250 m	X		8/12/2012	Sweden	Norrbottens	stor palsgöl syd Háldimarrasat	Claes Kyrk | ART 42557932, 42557933	GBIF
68.464496, 20.991436 ±100 m	X		8/10/2012	Sweden	Norrbottens	liten sjö SE Háldimarrasat	Claes Kyrk | ART 42557801	GBIF
68.466667, 20.898333	X		1994?	Sweden	Norrbottens	Lake Dávvajávri		Schröter, et al. 2012
68.47204, 20.980189 ±250 m	X		8/1/2012	Sweden	Norrbottens	Lilltundran, N Háldirmarrasat	Magnus Billqvist, Johan Lorentzon, Pav Johnsson, Magnus Billqvist, Christer Bergendorff, Robin Pranter, Ola Elleström | ART 42506750, 42506751	GBIF
68.47207, 20.982157 ±250 m	X		8/13/2012	Sweden	Norrbottens	Palsgölarna N Háldimarrasat	Claes Kyrk | ART 42557940, 42557941	GBIF
68.472606, 20.981255	X		2012	Sweden	Norrbottens	E Dávvajávri	Magnus Billqvist	Magnus Billqvist, pers. comm.
68.473431, 20.95554 ±100 m	X		8/11/2012	Sweden	Norrbottens	Palsgöl NE Dávvajávri	Claes Kyrk | ART 42557804	GBIF
68.474832, 20.971287 ±1000 m	X		7/30/2012	Sweden	Norrbottens	Pulsujärvi, norr Haldimarrasat, Karesua	Christer Bergendorff | ART 42499062	GBIF
68.477064, 21.007519	X		2012	Sweden	Norrbottens	E Dávvajávri	Magnus Billqvist	Magnus Billqvist, pers. comm.
68.478872, 20.968538 ±50 m	X		7/28/2011	Sweden	Norrbottens	ONO Dávvajávris nordspets	Magnus Billqvist, Ola Elleström | ART 42194522	GBIF
68.485403, 20.958028 ±100 m	X		7/28/2011	Sweden	Norrbottens	150 m N lilla sjön	Magnus Billqvist, Ola Elleström | ART 42194564	GBIF
68.489218, 20.933674	X		1994	Sweden	Norrbottens	Near Lake Dávvajávri		Sahlén 1994
68.495241, 21.01199 ±1000m	X		7/30/2015	Sweden	Norrbottens	Pulsujärvi, norr Haldimarrasat	Claes Möllersten, Lars Rigbäck, Stefan Lithner, Leif Tägtström, Lars G Petersson | ART 42498930	GBIF
68.669079, 89.378723	E			Russia	Krasnoyarsk Krai	Northern Siberia		Schröter 2011, est. Fig. 4
68.6884, -134.1262	X		7/8/1987	Canada	NWT	Reindeer Station, pond A, S of riverside lake	SG Cannings | SEM 5280–5285	SGC
68.6884, -134.1262	X		7/8/1987	Canada	NWT	Reindeer Station, pond C, S of riverside lake	SG Cannings | SEM 5286–5291	SGC
68.691, -134.137	X		7/10/1948	Canada	NWT	Reindeer Station, probably in vicinity of the station	JR Vockeroth | CNC	SGC
68.691, -134.137	X		7/12/1948	Canada	NWT	Reindeer Station, probably in vicinity of the station	WJ Brown | CNC	SGC
68.691, -134.137	X		7/13/1948	Canada	NWT	Reindeer Station, probably in vicinity of the station	JR Vockeroth | CNC	SGC
68.691, -134.137	X		7/19/1948	Canada	NWT	Reindeer Station, probably in vicinity of the station	JR Vockeroth | CNC	SGC
68.75431, 134.4672	E		1973	Russia	Sakha Republic	Confluence of Bytantay River with Yana River		Belyshev 1973, est. Fig. 145
68.8041, -148.82355	X		6/28/2003	USA	AK	North Slope Co., Dalton HWY, mi marker 303 near Slope Mountain	John C Abbott | OC343781	Abbott 2006

Coordinates (in dec. deg.)	Coord. Type[a]	Elevation	Record Date (M/D/Y)	Country	State /Province	Locality Description[b]	Collector/Observer | Deposition Information[c]	Source[d]
68.876402, 21.00951 ±75m	X		7/25/2015	Sweden	Norrbottens	V Keinovuopio, palsmyr	Robin Pranter, Jesper Wadstein, Kajsa Svensson | ART 63131179	GBIF
68.916667, 85.964869	E		7/25/1876	Russia	Krasnoyarsk Krai	Siberia, Patapovskoje, along Yenisei River		Hämäläinen 2015, pp. 26-30
68.92286, 86.13831	E		1973	Russia	Krasnoyarsk Krai	Yenisei River near SW corner of Taymyrsky Dolgano-Nenetsky District		Belyshev 1973, est. Fig. 148
68.931639, 20.932653	X		2016	Finland	Lapland	Kilpisjärvi	Göran Sahlén	Mäkinen 2015
68.969909, 78.876784	E		1981	Russia	Yamalo-Nenets Autonomous Okrug	northern Siberia, peninsula Tajmyr [Таймыр] – Gydan [Гыдан]		Belyshev & Kharitonov 1981, via Holuša 2009
69.019509, 20.891361	X		7/31/2015	Finland	Lapland	Enontekiö, Tsahkaljoki, Kilpisjärvi	Hannu Eskonen	FinBIF, 2018
69.143991, 21.075832	X		7/14/2014	Finland	Lapland	Enontekiö, Urtasvaara	Männistö K, Savolainen P & Tiittanen J	FinBIF, 2018
69.168752, 21.410157	X		7/9/2007	Finland	Lapland	Enontekiö, Annjaloanji	Markus P. Rantala	FinBIF, 2018
69.215169, 85.65119	E		≤2011	Russia	Krasnoyarsk Krai	northern Siberia		Schröter 2011, est. Fig. 4
69.333296, 26.107613	X		7/12/1966	Finland	Lapland	Utsjoki, Karigasniemi	Matti Hämäläinen	FinBIF, 2018
69.346111, 26.233056	X		7/12/1966	Finland	Lapland	Utsjoki, Karigasniemi		Hämäläinen 1967
69.3669, -152.1439	E		7/16/1959	USA	AK	Umiat	R Madge | CNC	SGC
69.3724, -148.7005 ±5 km	X	250±50 m	7/15/1970	USA	AK	Sagwon	Richard Gorham | UAM 100027227, 100027237, 100027198	GBIF
69.3724, -148.7005 ±5 km	X	250±50 m	7/23/1970	USA	AK	Sagwon	Richard Gorham | UAM 100027206, 100027213, 100027260, 100027267	GBIF
69.416667, 86.129217	E		7/30/1876	Russia	Krasnoyarsk Krai	Siberia, Dudinka (Dudinskoje), along Yenisei River		Hämäläinen 2015, pp. 26-28
69.618472, 27.027295	X		7/24/2009	Finland	Lapland	Utsjoki, Pulmankijärventien lammet	Kari Hiilosma, Veli-Matti Jokela	FinBIF, 2018
69.618472, 27.027295	X		7/13/2010	Finland	Lapland	Utsjoki, Lampi Petsikontien varressa (E75) 1.5 km N Jorbajärventie	Asmus Schröter	FinBIF, 2018
69.680344, 30.743709	X		7/13/2009	Norway		Sørvaranger, Sør-Varanger, Fi	Eivind Sørnes | NEF: SO2-Bugs: 27457	GBIF
69.680344, 30.743709	X		8/22/2009	Norway		Sørvaranger, Sør-Varanger, Fi	Eivind Sørnes | NEF: SO2-Bugs: 47075	GBIF
69.680344, 30.743709	X		8/2/2011	Norway		Sørvaranger, Sør-Varanger, Fi	Eivind Sørnes | NEF: SO2-Bugs: 151085	GBIF
69.706324, 30.802969	X		2014	Norway		Easternmost Sør-Varanger	Hans Olsvik, Jessica Ware, Göran Sahlén	This study
69.707768, 30.794837	X		2014	Norway		Easternmost Sør-Varanger	Hans Olsvik, Jessica Ware, Göran Sahlén	This study
69.707823, 30.79515 ±30 (m?)	X	182 m	7/23/1992	Norway		dam 1 km Ø f. Holmvatn	Hans A. Olsvik | NTNU-VM: OFU: Odona10715	GBIF
69.708876, 30.785397 ±30 (m?)	X		7/23/1992	Norway		dam 300 m Ø f. Holmvatn	Hans A. Olsvik | NTNU-VM: OFU: Odona10718	GBIF
69.715437, 30.74572	X		2014	Norway		Easternmost Sør-Varanger	Hans Olsvik, Jessica Ware, Göran Sahlén	This study
69.715987, 30.52194 ±30 (m?)	X		7/23/1992	Norway		dam 1 N f. Vintervollvatn	Hans A. Olsvik | NTNU-VM: OFU: Odona10716	GBIF
69.716505, 30.523043 ±30 (m?)	X		7/23/1992	Norway		dam 2 N f. Vintervollvatn	Hans A. Olsvik | NTNU-VM: OFU: Odona10717	GBIF
69.717239, 30.5255	X		2014	Norway		Easternmost Sør-Varanger	Jessica Ware, Göran Sahlén	This study

Coordinates (in dec. deg.)	Coord. Type[a]	Elevation	Record Date (M/D/Y)	Country	State /Province	Locality Description[b]	Collector/Observer | Deposition Information[c]	Source[d]
69.735396, 29.829474 ±30 (m?)	X	85 m	7/30/1992	Norway		Høybuktmoen, tjern V f. Makkskjermyra	Hans A. Olsvik | NTNU-VM: OFU: Odona10720	GBIF
69.744167, 26.981944	X		1986	Finland	Lapland	Utsjoki, Kevo; same site where Sahlén first recorded S. sahlbergi in 1986	Göran Sahlén	This study
69.744816, 26.979311	X		1987	Finland	Lapland	Utsjoki, near Kevojärvi		Sahlén 1987
69.744849, 26.978714	X		7/24/1989	Finland	Lapland	Utsjoki, Kevo	Pekka Valtonen	FinBIF, 2018
69.761433, 27.008475	X		7/30/1988	Finland	Lapland	Utsjoki, Kevo	SG Butler	FinBIF, 2018
69.770383, 26.930771	X		7/31/1988	Finland	Lapland	Utsjoki, Kevo	SG Butler	FinBIF, 2018
69.779664, 30.817296 ±30 (m?)	X	40 m	7/23/1992	Norway		Grense Jakobselv, myrtjern v. Oscar II kapell	Hans A. Olsvik | NTNU-VM: OFU: Odona10719	GBIF
69.779713, 30.817045	X		2014	Norway		Easternmost Sør-Varanger	Hans Olsvik, Jessica Ware, Göran Sahlén	This study
69.783744, 26.866202	X		7/14/1986	Finland	Lapland	Utsjoki, Kevo	Göran Sahlén	FinBIF, 2018
69.783744, 26.866202	X		7/29/1988	Finland	Lapland	Utsjoki, Kevo	SG Butler	FinBIF, 2018
69.783744, 26.866202	X		7/25/2009	Finland	Lapland	Utsjoki, Kevo	Matti Hämäläinen, Sami Karjalainen	FinBIF, 2018
69.783751, 27.125391	X		8/1/1988	Finland	Lapland	Utsjoki, Kevo	SG Butler	FinBIF, 2018
69.803497, 26.997394	X		≤2011	Finland	Lapland	Utsjoki		Schröter 2011
69.989417, 29.485269	X		≤2011	Norway		W Bugoynes		Schröter 2011
70.003935, 29.252562	X		7/7/1990	Norway		Brannsletta	Henning Pedersen | NTNU-VM: OFU: Odona10304	GBIF
70.004625, 29.250614	X	81 m	7/29/2001	Norway		Finnmark, Sør-Varanger, near Gandvik, approx. 5 km E of the settlement	Otakar Holuša	Holuša, 2009
70.050278, 27.88	X		7/23/2000	Finland	Lapland	Utsjoki, Nuorgam	Göran Sahlén	This study
70.050363, 27.914547	X		8/5/2008	Finland	Lapland	Utsjoki, Nuorgam	Petro Pynnönen	FinBIF, 2018
70.050363, 27.914547	X		7/23/2009	Finland	Lapland	Utsjoki, Nuorgam	Sami Karjalainen, Matti Hämäläinen	FinBIF, 2018
70.050363, 27.914547	X		8/5/2009	Finland	Lapland	Utsjoki, Nuorgam	Petteri Mäkelä, Teppo Lehtola, Risto Vilen	FinBIF, 2018
70.050363, 27.914547	X		7/21/2010	Finland	Lapland	Utsjoki, Nuorgam	Dietmar Ikemeyer, Matthias Olthoff	FinBIF, 2018
70.050363, 27.914547	X		7/24/3009	Finland	Lapland	Utsjoki, Nuorgam	Sami Karjalainen, Matti Hämäläinen	FinBIF, 2018
70.053611, 27.880278	X	195 m	7/23-24/2009 & 7/22-28/2011	Finland	Lapland	Utsjoki, fjell high plateau, open tundra		Schröter, et al. 2012
70.054266, 27.881493	X		7/10/2006	Finland	Lapland	Utsjoki, Nuorgam	Janne Koskinen	FinBIF, 2018
70.054338, 27.908411	X		7/30/2010	Finland	Lapland	Utsjoki, Nuorgam	Petri Metsälä, Petri Parkko, Jukka Toivanen	FinBIF, 2018
70.055091, 27.875084	X		7/26/2011	Finland	Lapland	Utsjoki, Nuorgam	Thomas Schneider, Elias Schneider	FinBIF, 2018
70.055091, 27.875084	X		7/27/2011	Finland	Lapland	Utsjoki, Nuorgam	Thomas Schneider, Elias Schneider	FinBIF, 2018
72.361439, 103.242139	E		≤2011	Russia	Krasnoyarsk Krai	northern Siberia		Schröter 2011, est. Fig. 4

[a] Coordinate types are: (X) exact coordinates given in a publication; or (E) coordinates that were estimated from map figures in a publication and/or textual descriptions, and by comparing them maps to those in Google Maps.

[b] Sections of the Dempster Highway (YT, Canada), were straightened after 2010, thus some kilometer marker numbers changed. Unless otherwise specified, Dempster Highway km markers should not be assumed to use the old or new numbering scheme.

[c] ART = Artportalen (Swedish Species Observation System), published by ArtDatabanken/Swedish Species Information Centre Sveriges Lantbruksuniversitet/Swedish University of Agricultural Sciences, Uppsala; CM = Carnegie Museum; CNC = Canadian National Collection of Insects, Arachnids, and Nematodes, Ottowa, ON; DTA = ?; JLW = Jessica L Ware Lab Collection, Biology Department, Rutgers University, Newark; OC =

OdonataCentral.org; RBCM = Royal BC Museum, Victoria; ROM = Royal ON Museum, Toronto; SEM = Spencer Entomological Collection, University of British Colombia, Vancouver; UAM = University of AK Museum of the North, Fairbainks

[d] GBIF = Global Biodiversity Information Facility (https://www.gbif.org/); SGC = Syd G Cannings, personal communication.

Supplementary Table S2. List of specimens used in phylogenetic reconstruction of *Somatochlora*.

Species/Tree ID	Source	Voucher No.	GenBank Accession No. (CO1)	GenBank Accession No. (D2)	Stage/Sex	Collection Date (M/D/Y)	Collection Locality	Locality Coordinates (in dec. deg.)	Collector
Cordulia amurensis									
CordAmu	GenBank		AB708895.1						
Helocorculia uhleri									
Uhleri1		Helocordulia uhleri1							
Uhleri2		Helocordulia uhleri							
Somatochlora albicincta									
AlbicinctaY1	This study	S38	MG847299	MG847339	larva	8/5/2015	Canada: YT: site code CA150805-01	66.268, -136.7765	MK Kohli, WR Kuhn
AlbicinctaY2	This study	S43	MG847300	MG847340	larva	8/5/2015	Canada: YT: site code CA150805-01	66.268, -136.7765	MK Kohli, WR Kuhn
AlbicinctaY3	This study	S39	MG847301	MG847341	larva	8/5/2015	Canada: YT: site code CA150805-01	66.268, -136.7765	MK Kohli, WR Kuhn
AlbicinctaY4	This study	S40	MG847302	MG847342	larva	8/5/2015	Canada: YT: site code CA150805-01	66.268, -136.7765	MK Kohli, WR Kuhn
AlbicinctaY5	This study	S44	MG847303	MG847343	larva	8/5/2015	Canada: YT: site code CA150805-01	66.268, -136.7765	MK Kohli, WR Kuhn
AlbicinctaY6	This study	S41	MG847304	MG847344	larva	8/5/2015	Canada: YT: site code CA150805-01	66.268, -136.7765	MK Kohli, WR Kuhn
AlbicinctaY7	This study	S37	MG847305	MG847345	larva	8/5/2015	Canada: YT: site code CA150805-03	66.2725, -136.7584	MK Kohli, WR Kuhn
AlbicinctaY8	This study	S35	MG847306	MG847346	larva	8/5/2015	Canada: YT: site code CA150805-03	66.2725, -136.7584	MK Kohli, WR Kuhn
AlbicinctaY9	This study	S36	MG847307	MG847347	larva	8/5/2015	Canada: YT: site code CA150805-03	66.2725, -136.7584	MK Kohli, WR Kuhn
AlbicinctaY10	This study	S76	MG847308	MG847348	larva	8/10/2015	Canada: YT: site code CA150810-01	66.78, -136.3407	MK Kohli, WR Kuhn
AlbicinctaY11	This study	S72	MG847309	MG847349	larva	8/10/2015	Canada: YT: site code CA150810-01	66.78, -136.3407	MK Kohli, WR Kuhn
AlbicinctaY13	This study	S58	MH560457		larva	8/12/2015	Canada: YT: site code CA150812-06	65.5919, -138.1697	MK Kohli, WR Kuhn
AlbicinctaY14	This study	S57	MH560458		larva	8/9/2015	Canada: YT: deep bog lake; site code CA150809-05	66.9324, -136.3228	MK Kohli, WR Kuhn
AlbicinctaY15	This study	S56	MH560460		larva	8/9/2015	Canada: YT: deep bog lake; site code CA150809-05	66.9324, -136.3228	MK Kohli, WR Kuhn
AlbicinctaY16	This study	S55	MG847310	MG847350	larva	8/9/2015	Canada: YT: deep bog lake; site code CA150809-05	66.9324, -136.3228	MK Kohli, WR Kuhn
AlbicinctaY17	This study	S51	MG847311	MG847351	larva	8/9/2015	Canada: YT: deep bog lake; site code CA150809-05	66.9324, -136.3228	MK Kohli, WR Kuhn
AlbicinctaY18	This study	S50	MG847312	MG847352	larva	8/9/2015	Canada: YT: deep bog lake; site code CA150809-05	66.9324, -136.3228	MK Kohli, WR Kuhn
AlbicinctaY19	This study	S49	MG847313	MG847353	larva	8/9/2015	Canada: YT: deep bog lake; site code CA150809-05	66.9324, -136.3228	MK Kohli, WR Kuhn
S. alpestris									
Alpestris1	GenBank		AB708912.1						
Alpestris2	GenBank		AB708911.1						
Alpestris3	GenBank		AB708910.1						
Alpestris4	GenBank		AB708909.1						
Alpestris5	GenBank		AB708908.1						
S. arctica									
Arctica1	GenBank		AB708913.1						
S. clavata									
Clavata1	GenBank		AB708916.1						
Clavata2	GenBank		AB708914.1						
Clavata3	GenBank		AB708915.1						
S. dido									
Dido1	GenBank		AB708918.1						
Dido2	GenBank		AB848397.1						
Dido3	GenBank		AB848399.1						
Dido4	GenBank		AB848398.1						
S. elongata									
Elongata1	GenBank		JN420263.1						

Species/Tree ID	Source	Voucher No.	GenBank Accession No. (CO1)	GenBank Accession No. (D2)	Stage/Sex	Collection Date (M/D/Y)	Collection Locality	Locality Coordinates (in dec. deg.)	Collector
Elongata2	GenBank		JN420262.1						
Elongata3	GenBank		KM528142.1						
Elongata4	GenBank		JN420264.1						
S. exuberata									
Exuberata1	GenBank		AB711468.1						
Exuberata2	GenBank		AB711467.1						
Exuberata3	GenBank		AB711466.1						
Exuberata4	GenBank		AB708919.1						
Exuberata5	GenBank		AB708924.1						
Exuberata6	GenBank		AB708922.1						
Exuberata7	GenBank		AB708923.1						
Exuberata8	GenBank		AB708920.1						
Exuberata9	GenBank		AB708921.1						
S. franklini									
Franklini1	GenBank		KM534638.1						
Franklini2	GenBank		KM531645.1						
FrankliniEP1	Erik Pilgrim	frank_SomfrA01_COI	MG874111		adult male	8/8/2001	Canada: BC: Mackenzie	55.685, -123.4578	
FrankliniEP2	Erik Pilgrim	frank_SomfrA02_COI	MG874112		adult male	7/7/2004	Canada: YT: Whitehorse	60.7939, -135.1681	
FrankliniEP3	Erik Pilgrim	frank_ODBOL-EPA1219_COI	MG874113		adult male	7/21/2009	Canada: YT: Dempster HWY, Ogilvie River	65.4218, -138.22	S&R Cannings
FrankliniEP4	Erik Pilgrim	frank_ODBOL-EPA1218_COI	MG874114		adult female	7/29/2009	Canada: YT: Whitehorse, Copper Ridge Fen	60.6941, -135.1189	S&R Cannings
FrankliniEP5	Erik Pilgrim	frank_ODBOL-EPA1215_COI	MG874115		adult male	7/29/2009	Canada: YT: Whitehorse, Copper Ridge Fen	60.6941, -135.1189	S&R Cannings
FrankliniEP6	Erik Pilgrim	frank_ODBOL-EPA1220_COI	MG874116		adult male	7/21/2009	Canada: YT: Dempster HWY, Ogilvie River	65.4218, -138.22	S&R Cannings
FrankliniEP7	Erik Pilgrim	frank_ODBOL-EPA1363_COI	MG874117		adult male	8/8/2010	Canada: YT: Whitehorse, Copper Ridge Fen	60.6941, -135.1189	S Cannings
FrankliniEP8	Erik Pilgrim	frank_ODBOL-EPA1398_COI	MG874118		adult male	8/12/2001	Canada: BC: Fort St. James, Carrier Lake	54.6331, -123.8139	R Cannings
FrankliniEP9	Erik Pilgrim	frank_ODBOL-EPA1400_COI	MG874119		adult male	6/20/2007	Canada: YT: 60 Mile Creek	64.0304, -140.7606	G Hutchings
FrankliniEP10	Erik Pilgrim	frank_ODBOL-EPA1364_COI	MG874120		adult female	7/29/2009	Canada: YT: Whitehorse, Copper Ridge Fen	60.6941, -135.1189	S Cannings
FrankliniEP11	Erik Pilgrim	frank_ODBOL-EPA1399_COI	MG874121		adult male	6/21/2007	Canada: YT: Klondike HWY	64.0158, -138.7878	G Hutchings
FrankliniEP12	Erik Pilgrim	frank_ODBOL-EPA1401_COI	MG874122		adult male	7/10/2005	Canada: YT: Cameron's Fen	60.1095, -125.8498	C Eckert
FrankliniEP13	Erik Pilgrim	frank_ODBOL-EPA1217_COI	MG874123		adult male	7/29/2009	Canada: YT: Whitehorse, Copper Ridge Fen	60.6941, -135.1189	S&R Cannings
FrankliniEP14	Erik Pilgrim	frank_ODBOL-EPA1216_COI	MG874124		adult male	7/29/2009	Canada: YT: Whitehorse, Copper Ridge Fen	60.6941, -135.1189	S&R Cannings
S. graeseri									
Graeseri1	GenBank		KF257081.1						
Graeseri2	GenBank		AB708930.1						
Graeseri3	GenBank		AB708928.1						
Graeseri4	GenBank		AB708927.1						
Graeseri5	GenBank		AB708926.1						
Graeseri6	GenBank		AB708925.1						
Graeseri7	GenBank		AB708929.1						
S. hudsonica									
HudsonicaEP1	Erik Pilgrim	hud_Somhud02_COI	MG874092		adult male	7/14/2003	Canada: BC: Shaker Lake	59.8231, -133.9183	
HudsonicaEP2	Erik Pilgrim	hud_Somhud04_COI	MG874093		adult male	7/15/2005	Canada: YT: Beaver Creek	62.5344, -140.1578	
HudsonicaEP3	Erik Pilgrim	hud_Somhud03_COI	MG874094		adult male	6/17/2003	Canada: YT: Whitehorse	60.585, -134.9092	
HudsonicaEP4	Erik Pilgrim	hud_Somhud01_COI	MG874095		adult male	8/10/2001	Canada: BC: Mackenzie	54.9903, -123.7339	

Species/Tree ID	Source	Voucher No.	GenBank Accession No. (CO1)	GenBank Accession No. (D2)	Stage/Sex	Collection Date (M/D/Y)	Collection Locality	Locality Coordinates (in dec. deg.)	Collector
HudsonicaEP5	Erik Pilgrim	hud_ODBOL-EPA1221_COI	MG874096		adult male	7/22/2009	Canada: YT: Dempster HWY, Eagle River	66.4461, -136.6913	S&R Cannings
HudsonicaEP6	Erik Pilgrim	hud_ODBOL-EPA1367_COI	MG874097		adult male	6/24/2010	Canada: YT: Upper Laird, Tanager Creek	60.0911, -128.9766	S Cannings
HudsonicaEP7	Erik Pilgrim	hud_ODBOL-EPA1369_COI	MG874098		adult male	6/26/2010	Canada: YT: Rancheria Mt.	60.1854, -129.918	S Cannings
HudsonicaEP8	Erik Pilgrim	hud_ODBOL-EPA1368_COI	MG874099		adult male	6/24/2010	Canada: YT: Upper Laird, Tanager Creek	60.0911, -128.9766	S Cannings
HudsonicaEP9	Erik Pilgrim	hud_ODBOL-EPA1365_COI	MG874100		adult male	6/24/2010	Canada: YT: Upper Laird, Tanager Creek	60.0911, -128.9766	S Cannings
HudsonicaEP10	Erik Pilgrim	hud_ODBOL-EPA1403_COI	MG874101		adult male	7/5/2005	Canada: YT: La Biche River	60.074, -124.0393	C Eckert
HudsonicaEP11	Erik Pilgrim	hud_ODBOL-EPA1404_COI	MG874102		adult male	6/20/2006	Canada: YT: Tthe Ndu Lake	62.7227, -136.6996	C Eckert
HudsonicaEP12	Erik Pilgrim	hud_ODBOL-EPA1406_COI	MG874103		adult male	7/7/2010	USA: AK	63.781, -145.7505	E Neipert
HudsonicaEP13	Erik Pilgrim	hud_ODBOL-EPA1366_COI	MG874104		adult male	6/24/2010	Canada: YT: Upper Laird, Tanager Creek	60.0911, -128.9766	S Cannings
HudsonicaEP14	Erik Pilgrim	hud_ODBOL-EPA1405_COI	MG874105		adult male	7/8/2010	USA: AK	63.781, -145.7505	E Neipert
HudsonicaEP15	Erik Pilgrim	hud_ODBOL-EPA1692_COI	MG874106		adult male	7/8/2011	USA: AK: Treeline Pond	63.781, -145.7505	
HudsonicaEP16	Erik Pilgrim	hud_ODBOL-EPA1693_COI	MG874107		adult male	7/20/2011	USA: AK: Treeline Pond	63.781, -145.7505	
HudsonicaEP17	Erik Pilgrim	hud_ODBOL-EPA1370_COI	MG874108		adult male	6/26/2010	Canada: YT: Rancheria Mt.	60.1854, -129.918	S Cannings
HudsonicaEP18	Erik Pilgrim	hud_ODBOL-EPA1691_COI	MG874109		adult male	7/8/2011	USA: AK: Treeline Pond	63.781, -145.7505	
HudsonicaEP19	Erik Pilgrim	hud_ODBOL-EPA1402_COI	MG874110		adult male	7/1/2008	Canada: BC: Boucher Lake	55.9902, -121.6632	R Cannings
S. metallica									
Metallica1	GenBank		AB708932.1						
Metallica2	GenBank		AB708931.1						
S. minor									
Minor1	GenBank		JN420265.1						
S. sahlbergi									
SahlbergiEP1	Erik Pilgrim	SAhl_SomsAh01_COI	MG874050		adult male	7/17/2005	Canada: YT: Dempster Highway	65.0619, -138.1261	
SahlbergiEP2	Erik Pilgrim	SAhl_ODBOL-EPA1237_COI	MG874051		adult male	7/27/2009	Canada: YT: Dempster HWY, Blackstone River	65.0638, -138.1279	S&R Cannings
SahlbergiEP3	Erik Pilgrim	SAhl_ODBOL-EPA1238_COI	MG874052		adult male	7/27/2009	Canada: YT: Dempster HWY, Blackstone River	65.0638, -138.1279	S&R Cannings
SahlbergiEP4	Erik Pilgrim	SAhl_SomsAh03_COI	MG874053		adult male	7/17/2005	Canada: YT: Dempster Highway	65.0619, -138.1261	
SahlbergiEP5	Erik Pilgrim	SAhl_SomsAh02_COI	MG874054		adult male	7/17/2005	Canada: YT: Dempster Highway	65.0619, -138.1261	
SahlbergiEP6	Erik Pilgrim	SAhl_ODBOL-EPA1234_COI	MG874055		adult male	7/27/2009	Canada: YT: Dempster HWY, Blackstone River	65.0638, -138.1279	S&R Cannings
SahlbergiEP7	Erik Pilgrim	SAhl_ODBOL-EPA1232_COI	MG874056		adult male	7/25/2009	Canada: YT: Dempster HWY, Engineer Creek	65.2069, -138.3249	S&R Cannings
SahlbergiEP8	Erik Pilgrim	SAhl_ODBOL-EPA1233_COI	MG874057		adult male	7/27/2009	Canada: YT: Dempster HWY, Engineer Creek	65.2069, -138.3249	S&R Cannings
SahlbergiEP9	Erik Pilgrim	SAhl_ODBOL-EPA1230_COI	MG874058		adult male	7/21/2009	Canada: YT: Dempster HWY, Ogilvie River	65.4272, -138.2267	S&R Cannings
SahlbergiEP10	Erik Pilgrim	SAhl_ODBOL-EPA1235_COI	MG874059		adult male	7/27/2009	Canada: YT: Dempster HWY, Blackstone River	65.0638, -138.1279	S&R Cannings
SahlbergiEP11	Erik Pilgrim	SAhl_ODBOL-EPA1241_COI	MG874060		adult male	7/27/2009	Canada: YT: Dempster HWY, Blackstone River	65.0638, -138.1279	S&R Cannings
SahlbergiEP12	Erik Pilgrim	SAhl_ODBOL-EPA1236_COI	MG874061		adult male	7/27/2009	Canada: YT: Dempster HWY, Blackstone River	65.0638, -138.1279	S&R Cannings
SahlbergiEP13	Erik Pilgrim	SAhl_ODBOL-EPA1229_COI	MG874062		adult male	7/21/2009	Canada: YT: Dempster HWY, Engineer Creek	65.2069, -138.3249	S&R Cannings
SahlbergiEP14	Erik Pilgrim	SAhl_ODBOL-EPA1409_COI	MG874063		adult female	6/17/2010	USA: AK	63.781, -145.7505	E Neipert
SahlbergiEP15	Erik Pilgrim	SAhl_ODBOL-EPA1408_COI	MG874064		adult male	7/7/2010	USA: AK	63.781, -145.7505	E Neipert
SahlbergiEP16	Erik Pilgrim	SAhl_ODBOL-EPA1240_COI	MG874065		adult male	7/27/2009	Canada: YT: Dempster HWY, Blackstone River	65.0638, -138.1279	S&R Cannings
SahlbergiEP17	Erik Pilgrim	SAhl_ODBOL-EPA1242_COI	MG874066		adult male	7/27/2009	Canada: YT: Dempster HWY, Ogilive River	65.4272, -138.2267	S&R Cannings
SahlbergiEP18	Erik Pilgrim	SAhl_ODBOL-EPA1231_COI	MG874067		adult male	7/24/2009	Canada: YT: Dempster HWY	65.6413, -138.1404	S&R Cannings
SahlbergiEP19	Erik Pilgrim	SAhl_ODBOL-EPA1239_COI	MG874068		adult female	7/27/2009	Canada: YT: Dempster HWY, Blackstone River	65.0638, -138.1279	S&R Cannings
SahlbergiEP20	Erik Pilgrim	SAhl_ODBOL-EPA1410_COI	MG874069		adult male	7/8/2010	USA: AK	63.781, -145.7505	E Neipert
SahlbergiEU1_Norway	This study	S10	MG847280	MG847320	larva	9/10/2014	Norway: Finnmark, Varanger, along HWY 890: Lake/pond in valley fed by small river/creek	69.7157, 30.7459	G. Sahlén & J.L. Ware

Species/Tree ID	Source	Voucher No.	GenBank Accession No. (CO1)	GenBank Accession No. (D2)	Stage/Sex	Collection Date (M/D/Y)	Collection Locality	Locality Coordinates (in dec. deg.)	Collector
SahlbergiEU2_Norway	This study	S9	MG847281	MG847321	larva	9/10/2014	Norway: Finnmark, Varanger, along HWY 889: Lake/pond in valley fed by small river/creek	69.7157, 30.7459	G. Sahlén & J.L. Ware
SahlbergiEU3_Norway	This study	S4	MG847282	MG847322	larva	9/10/2014	Norway: Finnmark, Varanger, along HWY 886: Small Lake/bok near road, w. seeping water	68.7079, 30.7952	G. Sahlén & J.L. Ware
SahlbergiEU4_Sweden	This study	S1	MG847283	MG847323	larva	9/7/2014	Sweden: Norrbottens län, Kiruna kommun, Pulsujärvi: Salix marsh, lots of mosses, seeping water	68.4532, 21.0399	G. Sahlén & J.L. Ware
SahlbergiEU5_Finland	This study	S12	MG847284	MG847324	larva	9/11/2014	Finland: Lapin maakunta, Kitillä, 15 km NE village: Reed lake lbeside road with small moss-filled pools	67.7457, 25.2343	G. Sahlén & J.L. Ware
SahlbergiEU6_Norway	This study	S8	MG847285	MG847325	larva	9/10/2014	Norway: Finnmark, Varanger, along HWY 888: Lake/pond in valley fed by small river/creek	69.7157, 30.7459	G. Sahlén & J.L. Ware
SahlbergiEU7_Norway	This study	S20	MH560456		larva	9/10/2014	Norway: Finnmark, Varanger, along HWY 891: Lake/pond in valley fed by small river/creek	69.7157, 30.7459	G. Sahlén & J.L. Ware
SahlbergiEU8_Norway	This study	S6	MG847286	MG847326	larva	9/10/2014	Norway: Finnmark, Varanger, along HWY 886: Lake/pond in valley fed by small river/creek	69.7157, 30.7459	G. Sahlén & J.L. Ware
SahlbergiEU9_Sweden	This study	S5	MG847287	MG847327	larva	9/8/2014	Sweden: Norrbottens län, Kiruna kommun, Pulsujärvi: Salix bog fed by river	68.4522, 21.0263	G. Sahlén & J.L. Ware
SahlbergiEU10_Norway	This study	S7	MG847288	MG847328	larva	9/10/2014	Norway: Finnmark, Varanger, along HWY 887: Lake/pond in valley fed by small river/creek	69.7157, 30.7459	G. Sahlén & J.L. Ware
SahlbergiEU11_Sweden	This study	S2	MG847289	MG847329	larva	9/7/2014	Sweden: Norrbottens län, Kiruna kommun, Pulsujärvi: Iron rich spring, sedges	68.4483, 21.0342	G. Sahlén & J.L. Ware
SahlbergiEU12_Finland	This study	S13	MG847290	MG847330	larva	9/11/2014	Finland: Lapin maakunta, Kitillä, 15 km NE village: Reed lake lbeside road with small moss-filled pools	67.7457, 25.2343	G. Sahlén & J.L. Ware
SahlbergiEU13_Finland	This study	S14	MG847291	MG847331	larva	9/11/2014	Finland: Lapin maakunta, Kitillä, 15 km NE village: Reed lake lbeside road with small moss-filled pools	67.7457, 25.2343	G. Sahlén & J.L. Ware
SahlbergiEU14_Norway	This study	S15	MG847292	MG847332	larva	9/10/2014	Norway: Finnmark, Varanger, along HWY 886: 5 ponds along road, shallow water, bedrock bottom, limited vegetation but some mosses	69.7169, 30.524	G. Sahlén & J.L. Ware
SahlbergiEU15_Norway	This study	S16			larva	9/10/2014	Norway: Finnmark, Varanger, along HWY 887: Small lake N of road fed by small river/creek and seeping water	69.7065, 30.8034	G. Sahlén & J.L. Ware
SahlbergiEU16_Norway	This study	S17	MG847293	MG847333	larva	9/10/2014	Norway: Finnmark, Varanger, along HWY 888: Lake/pond in valley fed by small river/creek	69.7157, 30.7459	G. Sahlén & J.L. Ware
SahlbergiEU17_Norway	This study	S18	MG847294	MG847334	larva	9/10/2014	Norway: Finnmark, Varanger, along HWY 889: Lake/pond in valley fed by small river/creek	69.7157, 30.7459	G. Sahlén & J.L. Ware
SahlbergiEU18_Norway	This study	S19	MG847295	MG847335	larva	9/10/2014	Norway: Finnmark, Varanger, along HWY 890: Lake/pond in valley fed by small river/creek	69.7157, 30.7459	G. Sahlén & J.L. Ware
SahlbergiEU19_Norway	This study	S21	MG847296	MG847336	larva	9/10/2014	Norway: Finnmark, Varanger, along HWY 892: Lake/pond in valley fed by small river/creek	69.7157, 30.7459	G. Sahlén & J.L. Ware
SahlbergiEU20_Norway	This study	S22	MG847297	MG847337	larva	9/10/2014	Norway: Finnmark, Varanger, along HWY 893: Lake/pond in valley fed by small river/creek	69.7157, 30.7459	G. Sahlén & J.L. Ware
SahlbergiEU21_Norway	This study	S23	MG847298	MG847338	larva	9/10/2014	Norway: Finnmark, Varanger, along HWY 894: Lake/pond in valley fed by small river/creek	69.7157, 30.7459	G. Sahlén & J.L. Ware
SahlbergiEU22_Finland	This study	Sahlbergi_Göran_Finalnd	MH560459				Finland	68.9153, 20.9737	Magnus Bilquist
SahlbergiNA1	This study (Beaty	S25	MG847274	MG847314	adult	9/15/2014	Canada: YT?		

Species/Tree ID	Source	Voucher No.	GenBank Accession No. (CO1)	GenBank Accession No. (D2)	Stage/Sex	Collection Date (M/D/Y)	Collection Locality	Locality Coordinates (in dec. deg.)	Collector
	Biodiversity Museum, UBC)								
SahlbergiNA2	This study (Beaty Biodiversity Museum, UBC)	S26	MG847275	MG847315	adult	9/15/2014	Canada: YT?		
SahlbergiNA3	This study (Beaty Biodiversity Museum, UBC)	S30	MG847276	MG847316	adult	7/10/1983	Canada: YT: Old Crow, ovipositing in Carex pond along SE side of airstrip	67.5734, -139.8234	RJ Cannings
SahlbergiNA4	This study	S32	MG847277	MG847317	adult	8/15/2015	Canada: YT: site code CA150815-05	65.0641, -138.128	MK Kohli, WR Kuhn
SahlbergiNA5	This study	S34	MG847278	MG847318	adult	8/15/2015	Canada: YT: site code CA150815-05	65.0641, -138.128	MK Kohli, WR Kuhn
SahlbergiNA6	This study	S33	MG847279	MG847319	adult	8/15/2015	Canada: YT: site code CA150815-05	65.0641, -138.128	MK Kohli, WR Kuhn
S. semicircularis									
Semicicularis1	GenBank		KM529041.1						
S. septentrionalis									
SeptrentionalisEP2	Erik Pilgrim	sep_ODBOL-EPA1226_COI	MG874076		adult male	7/29/2009	Canada: YT: Whitehorse, Copper Ridge Fen	60.6941, -135.1189	S&R Cannings
SeptrentionalisEP3	Erik Pilgrim	sep_ODBOL-EPA1224_COI	MG874077		adult male	7/4/2009	Canada: YT: Craig Lake Fen	60.2692, -134.4351	S&R Cannings
SeptrentionalisEP4	Erik Pilgrim	sep_ODBOL-EPA1227_COI	MG874078		adult male	7/29/2009	Canada: YT: Whitehorse, Copper Ridge Fen	60.6941, -135.1189	S&R Cannings
SeptrentionalisEP5	Erik Pilgrim	sep_ODBOL-EPA1228_COI	MG874079		adult male	7/29/2009	Canada: YT: Whitehorse, Copper Ridge Fen	60.6941, -135.1189	S&R Cannings
SeptrentionalisEP6	Erik Pilgrim	sep_ODBOL-EPA1225_COI	MG874080		adult male	7/29/2009	Canada: YT: Whitehorse, Copper Ridge Fen	60.6941, -135.1189	S&R Cannings
SeptrentionalisEP7	Erik Pilgrim	sep_ODBOL-EPA1375_COI	MG874081		adult male	7/18/2006	Canada: YT: Whitehorse, Copper Ridge Fen	60.6971, -135.1183	R Cannings
SeptrentionalisEP8	Erik Pilgrim	sep_ODBOL-EPA1418_COI	MG874082		adult male	6/21/2004	Canada: YT: Marsh Lake	60.4297, -134.2386	C Eckert
SeptrentionalisEP9	Erik Pilgrim	sep_ODBOL-EPA1419_COI	MG874083		adult male	6/17/2005	Canada: YT: Kookatsoon Lake	60.5537, -134.8746	C Eckert
SeptrentionalisEP10	Erik Pilgrim	sep_ODBOL-EPA1374_COI	MG874084		adult male	7/17/2006	Canada: YT: Craig Lake Fen	60.2635, -134.4348	R Cannings
SeptrentionalisEP11	Erik Pilgrim	sep_ODBOL-EPA1376_COI	MG874085		adult male	8/8/2010	Canada: YT: Whitehorse, Copper Ridge Fen	60.6941, -135.1189	S Cannings
SeptrentionalisEP12	Erik Pilgrim	sep_ODBOL-EPA1371_COI	MG874086		adult male	8/17/2008	Canada: YT: Whitehorse, Copper Ridge Fen	60.6937, -135.1188	S Cannings
SeptrentionalisEP13	Erik Pilgrim	sep_ODBOL-EPA1373_COI	MG874087		adult male	6/19/2010	Canada: YT: Craig Lake Fen	60.2667, -134.4365	S Cannings
SeptrentionalisEP14	Erik Pilgrim	sep_Somsep02_COI	MG874088		adult male	7/19/2003	Canada: BC: Tutshi River	59.7819, -134.9175	
SeptrentionalisEP15	Erik Pilgrim	sep_Somsep01_COI	MG874089		adult male	6/25/2004	Canada: YT: Judas Creek		
SeptrentionalisEP16	Erik Pilgrim	sep_ODBOL-EPA1417_COI	MG874090		adult male	7/14/2003	Canada: BC: Jones Lake	59.8581, -133.9142	S Cannings
SeptrentionalisEP17	Erik Pilgrim	sep_ODBOL-EPA1372_COI	MG874091		adult male	8/5/2009	Canada: YT: Whitehorse, Copper Ridge Fen	60.6941, -135.1189	S Cannings
S. uchidai									
Uchidai1	GenBank		AB708936.1						
Uchidai2	GenBank		AB708935.1						
Uchidai3	GenBank		AB708933.1						
Uchidai4	GenBank		AB708934.1						
S. viridiaenea									
Viridiaenea1	GenBank		AB708938.1						
Viridiaenea2	GenBank		AB708937.1						
S. whitehousei									
WhitehouseiEP1	Erik Pilgrim	white_Somwhi02_COI	MG874070		adult male	7/10/2004	Canada: YT: Halfway Lakes	63.7936, -135.815	

Species/Tree ID	Source	Voucher No.	GenBank Accession No. (CO1)	GenBank Accession No. (D2)	Stage/Sex	Collection Date (M/D/Y)	Collection Locality	Locality Coordinates (in dec. deg.)	Collector
WhitehouseiEP2	Erik Pilgrim	white_Somwhi01_COI	MG874071		adult male	8/14/2001	Canada: BC: Takla Landing	55.4808, -125.7036	
WhitehouseiEP3	Erik Pilgrim	white_ODBOL-EPA1420_COI	MG874072		adult male	7/10/2003	Canada: BC: Meziadin Junction	56.4081, -129.4217	G Hutchings
WhitehouseiEP4	Erik Pilgrim	white_ODBOL-EPA1424_COI	MG874073		adult female	8/8/2004	Canada: YT: Watson Lake	60.1747, -129.0964	G Hutchings
WhitehouseiEP5	Erik Pilgrim	white_ODBOL-EPA1427_COI	MG874074			7/17/2005	Canada: YT: Peel River	66.6775, -133.908	C Eckert
WhitehouseiEP6	Erik Pilgrim	white_ODBOL-EPA1422_COI	MG874075		adult male	8/11/2001	Canada: BC: Fort St. James, Tezzeron Creek	54.9019, -124.2389	R Cannings
S. williamsoni									
Williamsoni1	GenBank		KM531663.1						
***Somatochlora* sp.**									
SomatSP1	GenBank		JN420261.1						
SomatSP2	GenBank		JN420260.1						
SomatSP3	GenBank		JN420259.1						
SomatSP4	GenBank		JN420250.1						
SomatSP5	GenBank		JN420256.1						
SomatSP6	GenBank		JN420244.1						
SomatSP7	GenBank		JN420242.1						
SomatSP8	GenBank		JN420236.1						
SomatSP9	GenBank		JN420232.1						
SomatSP10	GenBank		JN420234.1						
SomatSP11	GenBank		JN420240.1						
SomatSP12	GenBank		JN420245.1						
SomatSP13	GenBank		JN420249.1						
SomatSP14	GenBank		JN420251.1						
SomatSP15	GenBank		JN420258.1						

Supplementary Table S3. List of primers.

Gene	Up Sequence	Up Source	Down Sequence	Down Source
COI	5'TAATTGGAGGA TTTGGAAATTG3'	Coi1709F, Wells and Sperling 1999	5'CCYGGTARAATTA RAATRTARACTTC3'	COI2191R, Simon et al. 1994
D2	5'TGCTTGAGAGTGCAGCCCAA3'	Ware et al. 2007	5'CCTTGGTCCGTGTTTCAAGAC3'	Ware et al. 2007

Supplementary Table S4. List of specimens used in haplotype networks.

Species	BOLD ID	GenBank ID	Collection Locality
Aeshna canadensis			
	BBEOD033-09		Canada: NS
	BBEOD036-09		Canada: NS
	BBEOD057-09		Canada: NS
	BBEOD058-09		Canada: NS
	BBODA268-10		Canada: SK
	CNBPP334-13		Canada: ON
	CNSLP1143-13		Canada: ON
	JLSOM016-08		Canada: ON
	JLSOM023-08		Canada: ON
	ODRMA205-10		Canada: BC
	ODRMA206-10		Canada: BC
	ODRMA207-10		Canada: BC
	ODRMA208-10		Canada: PE
	ODSO718-08		Canada: MB
	ODSO719-08		Canada: MB
	ODSO720-08		Canada: MB
	ODSO732-08		Canada: MB
	ODSO754-08		Canada: MB
	SDP648015-17		USA: ME
	SMTPO7905-15		Canada: NB
A. eremita			
	BBEOD022-09		Canada: NB
	BBEOD069-09		Canada: NL
	BBEOD083-09		Canada: NL
	BBEOD086-09		Canada: NL
	BBEOD088-09		Canada: NL
	BBEOD090-09		Canada: NL
	BBEOD133-09		Canada: NL
	BBEOD143-09		Canada: NL
	BBEOD151-09		Canada: NL
	BBODA052-10		Canada: SK
	BBODA053-10		Canada: SK
	BBODA054-10		Canada: SK
	BBODA061-10		Canada: SK
	BBODA064-10		Canada: SK
	BBODA065-10		Canada: SK
	BBODA066-10		Canada: SK
	BBODA325-10		Canada: BC
	BBODA329-10		Canada: BC
	BBODA352-10		Canada: AB
	CHLOC069-07		Canada: MB
	CHLOC076-07		Canada: MB
	OCAP016-07		Canada: MB
	ODRMA041-10		Canada: YT
	ODRMA042-10		Canada: YT
	ODRMA377-10		Canada: BC
	ODRMA378-10		Canada: BC
	ODSO658-08		Canada: AB
	ODSO659-08		Canada: AB
	ODSO707-08		Canada: MB
	ODSO708-08		Canada: MB
	ODSO709-08		Canada: MB
	ODSO710-08		Canada: MB
	ODSO711-08		Canada: MB
	ODSO713-08		Canada: MB
	ODSO714-08		Canada: MB
	ODSO727-08		Canada: MB
	ODSO731-08		Canada: MB
	ODSO780-08		Canada: MB
	ODSO792-08		Canada: ON
	ODSO815-08		Canada: ON
	UAMIC839-13		USA: AK
	UAMIC842-13		USA: AK
A. interrupta			
	BBEOD008-09		Canada: NB
	BBEOD029-09		Canada: NB
	BBEOD093-09		Canada: NL
	BBEOD141-09		Canada: NL
	BBEOD170-09		Canada: NB
	BBODA014-10		Canada: AB
	BBODA016-10		Canada: AB
	BBODA050-10		Canada: SK
	BBODA059-10		Canada: SK
	BBODA063-10		Canada: SK
	BBODA067-10		Canada: SK
	BBODA326-10		Canada: BC
	BBODA327-10		Canada: BC
	BBODA351-10		Canada: AB
	ODRMA070-10		Canada: YT
	ODRMA071-10		Canada: YT
	ODRMA132-10		Canada: YT
	ODRMA133-10		Canada: YT
	ODRMA213-10		Canada: PE
	ODSO304-08		Canada: MB
	ODSO310-08		Canada: MB
	ODSO311-08		Canada: MB
	ODSO312-08		Canada: MB
	ODSO313-08		Canada: MB
	ODSO350-08		Canada: MB
	ODSO351-08		Canada: MB
	ODSO352-08		Canada: MB
	ODSO398-08		Canada: MB
	ODSO491-08		Canada: AB
	ODSO511-08		Canada: AB
	ODSO622-08		Canada: AB
	ODSO623-08		Canada: AB
	ODSO625-08		Canada: AB
	ODSO626-08		Canada: AB
	ODSO627-08		Canada: AB
	ODSO628-08		Canada: AB
	ODSO632-08		Canada: AB
	ODSO649-08		Canada: AB
	ODSO655-08		Canada: AB
	ODSO664-08		Canada: AB
	ODSO671-08		Canada: AB
	ODSO672-08		Canada: AB
	ODSO677-08		Canada: AB
	ODSO678-08		Canada: AB
	ODSO712-08		Canada: MB
	ODSO715-08		Canada: MB
	ODSO717-08		Canada: MB
	ODSO728-08		Canada: MB
	ODSO729-08		Canada: MB
	ODSO730-08		Canada: MB
	ODSO838-08		Canada: ON
	ODSO839-08		Canada: ON
	ODSO840-08		Canada: ON
	ODSO841-08		Canada: ON
	UAMIC775-13		USA: AK
	UAMIC776-13		USA: AK
A. juncea			
	BBODA055-10		Canada: SK
	BBODA057-10		Canada: SK
	BBODA058-10		Canada: SK
	CHLOC009-07		Canada: MB
	CHLOC028-07		Canada: MB
	CHLOC029-07		Canada: MB

Species	BOLD ID	GenBank ID	Collection Locality
***A. juncea* (cont.)**			
	CHLOC030-07		Canada: MB
	CHLOC032-07		Canada: MB
	CHLOC033-07		Canada: MB
	CHLOC038-07		Canada: MB
	CHLOC054-07		Canada: MB
	CHLOC057-07		Canada: MB
	CHLOC064-07		Canada: MB
	CHLOC067-07		Canada: MB
	CHLOC070-07		Canada: MB
	CHLOC075-07		Canada: MB
	CHLOC080-07		Canada: MB
	CHLOC092-07		Canada: MB
	CHLOC106-07		Canada: MB
	CHLOC107-07		Canada: MB
	CHLOC110-07		Canada: MB
	CHLOC115-07		Canada: MB
	FBAQU1431-13		Germany: Bavaria
	FBAQU479-10		Germany: Bavaria
	FBAQU521-10		Germany: Bavaria
	GBMIN24091-13	AB711463	Russia
	GBMIN24424-13	AB708591	Russia
	GBMIN24425-13	AB708589	Japan: Hokkaido
	GBMIN24426-13	AB708587	Japan
	GBMIN24427-13	AB708585	Japan: Hokkaido
	GBMIN24428-13	AB708583	Russia
	GBMIN24429-13	AB708581	Japan: Hokkaido
	GBMIN24574-13	AB711464	South Korea
	GBMIN24577-13	AB711458	Finland
	GBMIN24909-13	AB708590	Japan: Hokkaido
	GBMIN24910-13	AB708588	Japan: Toyama
	GBMIN24911-13	AB708586	Russia
	GBMIN24912-13	AB708584	Japan: Hokkaido
	GBMIN24913-13	AB708582	Russia
	GSAC044-07		Canada: MB
	GSAC056-07		Canada: MB
	ODOPH001-13		Netherlands
	ODRMA036-10		Canada: YT
	ODRMA037-10		Canada: YT
	ODRMA038-10		Canada: YT
	ODRMA083-10		Canada: BC
	UAMIC785-13		USA: AK
	UAMIC837-13		USA: AK
A. septentrionalis			
	CHLOC040-07		Canada: MB
	CHLOC041-07		Canada: MB
	CHLOC042-07		Canada: MB
	CHLOC045-07		Canada: MB
	CHLOC046-07		Canada: MB
	CHLOC048-07		Canada: MB
	CHLOC049-07		Canada: MB
	CHLOC052-07		Canada: MB
	CHLOC074-07		Canada: MB
	CHLOC128-07		Canada: MB
	GSAC005-07		Canada: MB
	GSAC007-07		Canada: MB
	GSAC008-07		Canada: MB
	GSAC009-07		Canada: MB
	GSAC010-07		Canada: MB
	GSAC011-07		Canada: MB
	JLSOM036-08		Canada: MB
	JLSOM038-08		Canada: MB
	OCAP019-08		Canada: MB
	OCAP022-08		Canada: MB
	ODRMA066-10		Canada: YT
	ODRMA068-10		Canada: YT

Species	BOLD ID	GenBank ID	Collection Locality
	ODRMA069-10		Canada: YT
	ODRMA214-10		Canada: YT
	UAMIC786-13		USA: AK
	UAMIC787-13		USA: AK
A. subarctica			
	BBODA056-10		Canada: SK
	CHLOC065-07		Canada: MB
	CHLOC068-07		Canada: MB
	GBMIN24092-13	AB711461	Japan: Hokkaido
	GBMIN24093-13	AB711459	Japan: Hokkaido
	GBMIN24422-13	AB708595	Japan: Hokkaido
	GBMIN24575-13	AB711462	Finland
	GBMIN24576-13	AB711460	Japan: Hokkaido
	GBMIN88537-17	KU180298	Germany
	ODRMA039-10		Canada: YT
	ODRMA040-10		Canada: YT
	ODRMA134-10		Canada: YT
	ODRMA215-10		Canada: BC
	UAMIC788-13		USA: AK
	UAMIC789-13		USA: AK
A. umbrosa			
	BBEOD005-09		Canada: NB
	BBEOD084-09		Canada: NL
	BBEOD128-09		Canada: NL
	BBEOD164-09		Canada: NB
	BBEOD174-09		Canada: NB
	BBEOD189-09		Canada: NB
	BBGCO327-15		Canada: BC
	BBGCO332-15		Canada: BC
	BBGCO505-15		Canada: BC
	BBGCO629-15		Canada: BC
	BBGCO630-15		Canada: BC
	BBODA068-10		Canada: SK
	BBODA328-10		Canada: BC
	ECODB036-09		Canada: NB
	ECODB205-09		Canada: NB
	ECODB263-09		Canada: NB
	ECODB264-09		Canada: NB
	ECODB265-09		Canada: NB
	ECODB313-09		Canada: NB
	ECODB336-09		Canada: NB
	ECODB360-09		Canada: NB
	ECODB361-09		Canada: NB
	ECODB362-09		Canada: NB
	ECODB363-09		Canada: NB
	ECODB364-09		Canada: NB
	ECODB365-09		Canada: NB
	ECODB366-09		Canada: NB
	ECODB367-09		Canada: NB
	ECODB368-09		Canada: NB
	ECODB369-09		Canada: NB
	ECODB370-09		Canada: NB
	ECODB475-09		Canada: NB
	ECODB476-09		Canada: NB
	ECODB510-09		Canada: NB
	ECODB659-09		Canada: NB
	ECODN057-09		Canada: NB
	ECODN120-09		Canada: NB
	ECODN122-09		Canada: NB
	ECODN172-09		Canada: NB
	ECODN173-09		Canada: NB
	ECODN174-09		Canada: NB
	ECODN297-09		Canada: NB
	ECODN420-09		Canada: NB
	ECODN421-09		Canada: NB
	ECODN422-09		Canada: NB

Species	BOLD ID	GenBank ID	Collection Locality
***A. umbrosa* (cont.)**			
	ECODN423-09		Canada: NB
	ECODN424-09		Canada: NB
	ECODN425-09		Canada: NB
	ECODN426-09		Canada: NB
	ECODN454-09		Canada: NB
	ECODN496-09		Canada: NB
	ECODN568-09		Canada: NB
	ECODN670-09		Canada: NB
	ECODN671-09		Canada: NB
	ECODN764-09		Canada: NB
	ECODN765-09		Canada: NB
	ECODN766-09		Canada: NB
	ECODN767-09		Canada: NB
	ECODN768-09		Canada: NB
	ECODN792-09		Canada: NB
	ECODN793-09		Canada: NB
	ECODN794-09		Canada: NB
	ECODN840-09		Canada: NB
	ECODN893-09		Canada: NB
	ECODN894-09		Canada: NB
	ECODN895-09		Canada: NB
	ECODN896-09		Canada: NB
	ECODN934-09		Canada: NB
	JLSOM033-08		Canada: NB
	ODRMA081-10		Canada: BC
	ODRMA220-10		Canada: BC
	ODRMA221-10		Canada: BC
	ODSO755-08		Canada: MB
	ODSO812-08		Canada: ON
	ODSO813-08		Canada: ON
	ODSO814-08		Canada: ON
Leucorrhinia glacialis			
	DOOCJ018-08		Canada: ON
	DOOCJ019-08		Canada: ON
	DOOCJ020-08		Canada: ON
	DOOCJ021-08		Canada: ON
	DOOCJ022-08		Canada: ON
	DOOCJ023-08		Canada: ON
	DOOCJ024-08		Canada: ON
	DOOCJ025-08		Canada: ON
	DOOCJ026-08		Canada: ON
	DOOCJ027-08		Canada: ON
	ODRMA312-10		Canada: BC
	ODSO237-08		Canada: ON
	ODSO261-08		Canada: ON
	ODSO316-08		Canada: MB
	ODSO321-08		Canada: MB
	UAMIC795-13		USA: AK
Libellula quadrimaculata			
	BBEOD072-09		Canada: NL
	BBEOD081-09		Canada: NL
	BBEOD130-09		Canada: NL
	BBGCO386-15		Canada: BC
	BBGEN164-15		Canada: ON
	BBODA001-10		Canada: AB
	BBODA078-10		Canada: SK
	BBODA080-10		Canada: SK
	BBODA082-10		Canada: SK
	BBODA083-10		Canada: SK
	BBODA119-10		Canada: SK
	BBODA121-10		Canada: SK
	BBODA122-10		Canada: SK
	BBODA123-10		Canada: SK
	BIOAI210-14		Canada: AB
	BIOAI267-14		Canada: AB
	BIOAI437-14		Canada: AB
	DOOCJ029-08		Canada: ON
	DOOCJ031-08		Canada: ON
	DOOCJ032-08		Canada: ON
	DOOCJ033-08		Canada: ON
	ECODB078-09		Canada: NB
	FBAQU503-10		Germany: Bavaria
	GBMIN24228-13	AB708986	Japan
	GBMIN24711-13	AB708987	Japan: Hokkaido
	GBMIN24712-13	AB708985	Japan
	ODRMA029-10		Canada: YT
	ODRMA030-10		Canada: YT
	ODRMA098-10		Canada: BC
	ODRMA099-10		Canada: BC
	ODSO216-08		Canada: ON
	ODSO220-08		Canada: ON
	ODSO234-08		Canada: ON
	ODSO238-08		Canada: ON
	ODSO239-08		Canada: ON
	ODSO240-08		Canada: ON
	ODSO241-08		Canada: ON
	SWCHL534-15		Canada: ON
	UAMIC767-13		USA: AK
	UAMIC823-13		USA: AK
Sympetrum danae			
	GBMH5904-09	EU243891	Russia
	GBMH5905-09	EU243890	Russia
	GBMH5940-09	EU243914	Canada: YT
	GBMH5941-09	EU243913	Canada: YT
	GBMH5942-09	EU243912	Canada: YT
	GBMH5943-09	EU243911	Canada: YT
	GBMH5944-09	EU243910	
	GBMH5945-09	EU243909	USA: WA
	GBMH5946-09	EU243908	USA: WA
	GBMH5947-09	EU243907	USA: WA
	GBMH5948-09	EU243906	USA: WA
	GBMH5949-09	EU243905	USA: UT
	GBMH5950-09	EU243904	USA: UT
	GBMH5951-09	EU243903	USA: UT
	GBMH5952-09	EU243902	USA: UT
	GBMH5953-09	EU243901	USA: UT
	GBMH5954-09	EU243900	USA: UT
	GBMH5955-09	EU243899	USA: UT
	GBMH5956-09	EU243898	USA: UT
	GBMH5957-09	EU243897	USA: UT
	GBMH5958-09	EU243896	USA: UT
	GBMH5959-09	EU243895	USA: UT
	GBMH5960-10		USA: UT
	GBMH5961-09	EU243893	USA: UT
	GBMH5962-09	EU243892	USA: UT
	GBMH5963-09	EU243889	USA: OR
	GBMH5964-09	EU243888	USA: OR
	GBMH5965-09	EU243887	USA: OR
	GBMH5966-09	EU243886	USA: OR
	GBMH5967-09	EU243885	USA: OR
	GBMH5968-09	EU243884	Canada: NT
	GBMH5969-09	EU243883	Canada: NT
	GBMH5970-09	EU243882	USA: MI
	GBMH5971-09	EU243881	USA: MI
	GBMH5972-09	EU243880	USA: MI
	GBMH5973-09	EU243879	USA: MI
	GBMH5974-09	EU243878	USA: MI
	GBMH5975-09	EU243877	USA: MI
	GBMH5976-09	EU243876	USA: MI
	GBMH5977-09	EU243875	USA: MI
	GBMH5978-09	EU243874	USA: MI

Species	BOLD ID	GenBank ID	Collection Locality
***Sympetrum danae* (cont.)**			
	GBMH5979-09	EU243873	USA: MI
	GBMH5980-09	EU243872	Canada: BC
	GBMH5981-09	EU243871	Canada: BC
	GBMH5982-09	EU243870	Canada: BC
	GBMH5983-09	EU243869	Canada: BC
	GBMH5984-09	EU243868	Canada: BC
	GBMH5985-09	EU243867	Canada: BC
	GBMH5986-09	EU243866	Canada: BC
	GBMH5987-09	EU243865	Canada: BC
	GBMH5988-09	EU243864	Canada: BC
	GBMH5989-09	EU243863	Canada: BC
	GBMH5990-09	EU243862	Canada: BC
	GBMH5991-09	EU243861	Canada: BC
	GBMH5992-09	EU243860	Canada: BC
	GBMH5993-09	EU243859	Canada: BC
	GBMH5994-09	EU243858	Canada: BC
	GBMH5995-09	EU243857	Canada: BC
	GBMH5996-09	EU243856	Canada: AK
	GBMH5997-09	EU243855	Canada: AK
	GBMH5998-09	EU243854	Canada: YT
	GBMH5999-09	EU243853	Canada: YT
	GBMH6000-09	EU243852	Canada: YT
	GBMH6001-09	EU243851	Canada: YT
	GBMH6002-09	EU243850	Canada: YT
	GBMH6003-09	EU243849	Canada: YT
	GBMH6004-09	EU243848	Canada: YT
	GBMH6005-09	EU243847	Canada: YT
	GBMH6006-09	EU243846	
	GBMH6007-09	EU243845	Russia
	GBMH6008-09	EU243844	Russia: Chita Province
	GBMH6009-09	EU243843	Russia
	GBMH6010-09	EU243842	Russia
	GBMH6011-09	EU243841	Russia: Chita Province
	GBMH6012-09	EU243840	Russia: Chita Province
	GBMH6013-09	EU243839	Netherlands
	GBMH6014-09	EU243838	Netherlands
	GBMH6015-09	EU243837	Netherlands
	GBMH6016-09	EU243836	Ireland
	GBMH6017-09	EU243835	Ireland
	GBMH6018-09	EU243834	Ireland
	GBMH6019-09	EU243833	Ireland
	GBMH6020-09	EU243832	Ireland
	GBMH6021-09	EU243831	Ireland
	GBMH6022-09	EU243830	Belarus?
	GBMH6023-09	EU243829	Belarus?
	GBMH6024-09	EU243828	Canada: BC
	GBMH6025-09	EU243827	Canada: BC
	GBMH6026-09	EU243826	USA: AK
	GBMH6027-09	EU243825	USA: AK
	GBMH6028-09	EU243824	USA: AK
	GBMH6029-09	EU243823	USA: AK
	GBMH6030-09	EU243822	USA: AK
	GBMH6031-09	EU243821	USA: AK
	GBMH6032-09	EU243820	USA: AK
	GBMH6033-09	EU243819	USA: AK
	GBMH6034-09	EU243818	USA: AK
	GBMH6035-09	EU243817	USA: AK
	GBMH6036-09	EU243816	USA: AK
	GBMIN24160-13	AB709122	Japan
	GBMIN24161-13	AB709120	Japan: Hokkaido
	GBMIN24644-13	AB709121	Japan
	GBMIN39980-13	EU743616	Russia
	GBMIN39981-13	EU743614	Russia
	GBMIN39982-13	EU743615	Russia
	GBMIN39983-13	EU743613	Russia

Supplementary Table S5. Intraspecific variation in 12 Holarctic dragonfly species.

Species	Range	n	KP2[a]	JC[b]	Percentage[c]
Aeshna canadensis	Canada and US	20	0.005	0.005	0.5
A. eremita	Canada and US	42	0.003	0.003	0.3
A. interrupta	Canada and US	56	0.005	0.005	0.5
A. juncea	Holarctic	48	0.019	0.019	1.9
A. septentronalis	Canada and US	26	0.002	0.002	0.2
A. subarctica	Holarctic	15	0.022	0.022	2.2
A. umbrosa	Canada	76	0.007	0.007	0.7
Leucorrhinia glacialis	Canada and US	16	0.004	0.004	0.4
Libellula quadrimaculata	Holarctic	40	0.010	0.010	1.0
Somatochlora albicincta	Samples collected from YT	18	0.001	0.001	0.1
S. sahlbergi	Holarctic	51	0.001	0.001	0.1
Sympetrum danae	Holarctic	106	0.036	0.036	3.6

[a] KP2 = Kimura-2-parameter nucleotide evolution model.

[b] JC = Jukes-Cantor nucleotide evolution model.

[c] Values obtained from KP2 and JC nucleotide evolution model converted into percentage.

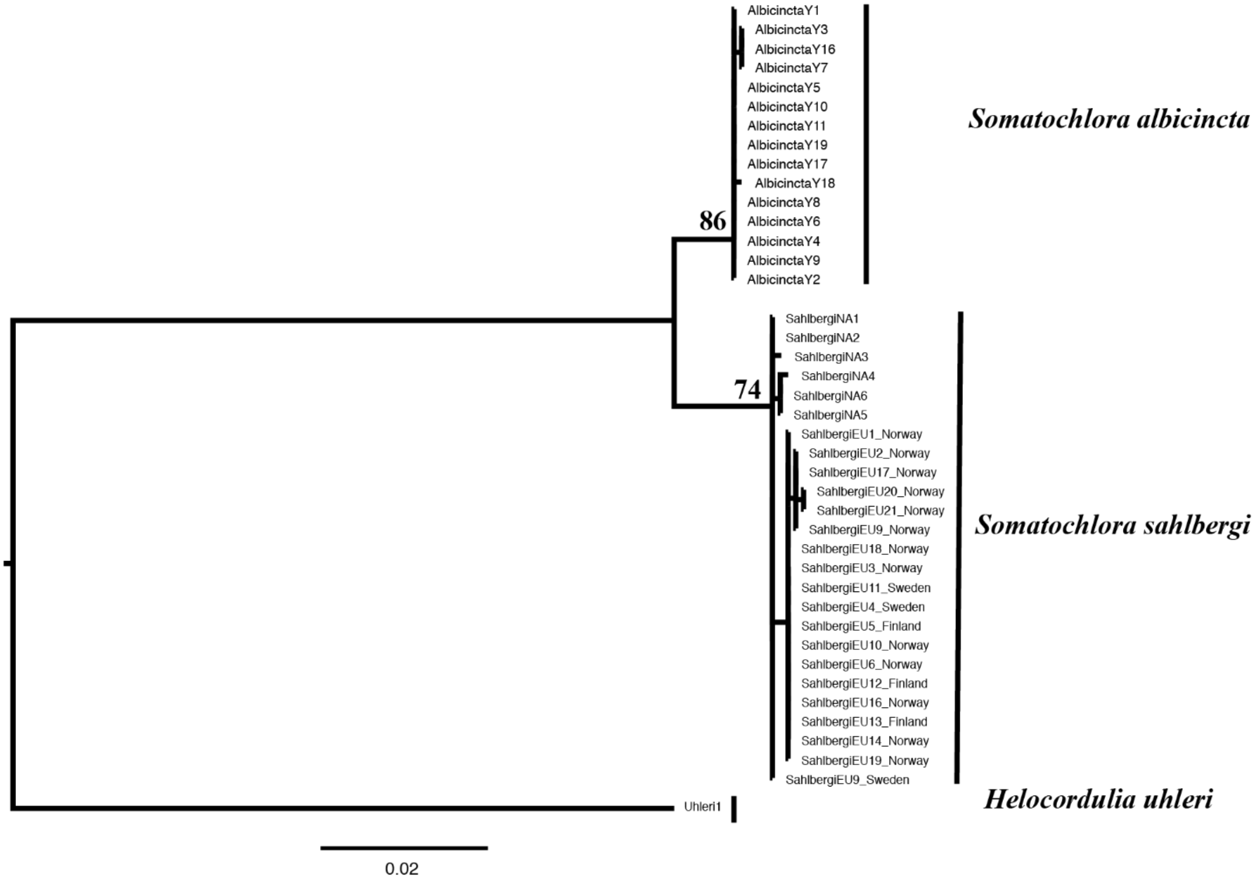

Supplementary Figure S1. COI+D2 Tree. *Somatochlora sahlbergi and S. albicincta* are recovered as monophyletic species. Within *S. sahlbergi*, all the individuals from North America and Europe are recovered in a polytomy. The numbers on the branches indicate the bootstrap values for the representative node.

References Cites in Supplementary Information

Abbott, J. C. OdonataCentral: An online resource for the distribution and identification of Odonata. (2006).

Belyshev, B. *The dragonflies of Siberia (Odonata).* **Vol. 1,** Part 2 (Nauka, 1973).

Belyshev, B. F. & Kharitonov, A. Y. *The Geography of Dragonflies (Odonata) of Boreal Faunistic Kingdom*. (Nauka, 1981).

Belyshev, B. F. & Ovodov, N. *Somatochlora sahlbergi* Trybom (Odonata, Insecta) in South Siberia. *Zoologičeskij Žurnal* **40,** 1892-1893 (1961).

Dumont, H. J., Haritonov, A. Y., Kosterin, O. & Popova, O. N. A review of the Odonata of Kamchatka Peninsula, Russia. *Odonatologica* **34,** 131-153 (2005).

Finnish Biodiversity Information Facility/FinBIF. http://tun.fi/HBF.30688 (accessed 2018-06-22).

Futashi, R. A revisional study of Japanese dragonflies based on DNA analysis. *Tombo* **53,** 67-74 (2012).

Hämäläinen, M. *Somatochlora sahlbergi* Trybom (Odon., Corduliidae) Utsjoelta (InL). *Luonnon Tutkija* **71,** 25 (1967).

Hämäläinen, M. The first collectors of *Somatochlora sahlbergi* – a story of an arduous expedition to Siberia in 1876. *Agrion* **20,** 22-31 (2015).

Holuša, O. The finding of *Somatochlora sahlbergi* (Odonata: Corduliidae) in the northern Norway. *Acta Musei Beskidensis* **1,** 97-102 (2009).

Kharitonov, A. Y. On the biology of little-known species of dragonflies *Somatochlora sahlbergi* Trybom (Odonata, Corduliidae). in *Animal taxonomy and ecology of animals of Siberia* (ed. Cherepanov, I. A.) 21-23 (Nauka, 1975).

Kharitonova, I. N. To the fauna of dragonflies (Insecta, Odonata) of mountains of South Siberia. in *Arthropods and Helminthes (Fauna of Siberia)* 43-47 (Nauka, 1990).

Sahlén, G. A new site for Somatochlora sahlbergi Trybom in Inari Lapland (Odonata, Corduliidae). *Notulae Entomol.* **67,** 3-4 (1987).

Schröter, A. *Review of the distribution of* Somatochlora sahlbergi *(Odonata: Corduliidae)*. 1-27 (International Dragonfly Fund, 2011).

Schröter, A., Schneider, T., Schneider, E., Karjalainen, S. & Hämäläinen, M. Observations on adult *Somatochlora sahlbergi* – a species at risk due to regional climate change? (Odonata: Corduliidae). *Libellula* **31,** 41-60 (2012).

Simon, C., Frati, F., Beckenbach, A., Crespi, B., Liu, H., & Flook, P. (1994). Evolution, weighting, and phylogenetic utility of mitochondrial gene sequences and a compilation of conserved polymerase chain reaction primers. *Ann. Entomol. Soc. Amer.* **87,** 651-701.

Wells, J. D., & Sperling, F. A. (1999). Molecular phylogeny of *Chrysomya albiceps* and *C. rufifacies* (Diptera: Calliphoridae). *J. Med. Entomol.* **36,** 222-226.

Степанов, Л. Н. Разнообразие зообентоса водоемов и водотоков бассейнов рек Сетная и Нгояха (полуостров Ямал, Ямало-Ненецкий автономный округ). *Fauna of the Urals and Siberia* **1,** 90-104 (2016).
